# Bispidine Platform as a Tool for Studying Amide Configuration Stability

**DOI:** 10.3390/molecules27020430

**Published:** 2022-01-10

**Authors:** Dmitry P. Krut’ko, Alexey V. Medved’ko, Konstantin A. Lyssenko, Andrei V. Churakov, Alexander I. Dalinger, Mikhail A. Kalinin, Alexey O. Gudovannyy, Konstantin Y. Ponomarev, Eugeny V. Suslov, Sergey Z. Vatsadze

**Affiliations:** 1 Chemistry Department, Lomonosov Moscow State University, Leninskie Gory, 119991 Moscow, Russia; kdp@org.chem.msu.ru (D.P.K.); klyssenko@gmail.com (K.A.L.); dal1995@mail.ru (A.I.D.); chem.kalinin@gmail.com (M.A.K.); alexeygudovannyy@gmail.com (A.O.G.); 2 Zelinsky Institute of Organic Chemistry, 119991 Moscow, Russia; lexeym@gmail.com; 3 N.S. Kurnakov Institute of General and Inorganic Chemistry, 119991 Moscow, Russia; churakov@igic.ras.ru; 4 High Chemical College, Mendeleev University of Chemical Technology of Russia, 125047 Moscow, Russia; 5 N.N. Vorozhtsov Novosibirsk Institute of Organic Chemistry, 630090 Novosibirsk, Russia; ponomarev@nioch.nsc.ru (K.Y.P.); suslov@nioch.nsc.ru (E.V.S.)

**Keywords:** 3,7-diazabicyclo[3.3.1]nonanes, dynamic stereochemistry, barriers of amide rotation, NMR spectroscopy, X-ray diffraction study, quantum chemical calculations

## Abstract

In this work, the solution conformations of seventeen 3,7-diacyl bispidines were studied by means of NMR spectroscopy including VT NMR experiments. The acyl groups included alkyl, alkenyl, aryl, hetaryl, and ferrocene moieties. The presence of syn/anti-isomers and their ratios were estimated, and some reasons explaining experimental facts were formulated. In particular, all aliphatic and heterocyclic units in the acylic R(CO) fragments led to an increased content of the *syn*-form in DMSO-*d*_6_ solutions. In contrast, only the *anti*-form was detected in DMSO-*d*_6_ and CDCl_3_ in the case when R = Ph, ferrocenyl, (*R*)-myrtenyl. In the case of a chiral compound derived from the natural terpene myrtene, a new dynamic process was found in addition to the expected inversion around the amide N-C(O) bond. Here, rotation around the CO-C=C bond in the acylic R fragment was detected, and its energy was estimated. For this compound, ΔG for amide N-C(O) inversion was found to be equal to 15.0 ± 0.2 kcal/mol, and for the rotation around the N(CO)–C^2′^ bond, it was equal to 15.6 ± 0.3 kcal/mol. NMR analysis of the chiral bispidine-based bis-amide was conducted for the first time. Two X-ray structures are reported. For the first time, the unique *syn*-form was found in the crystal of an acyclic bispidine-based bis-amide. Quantum chemical calculations revealed the unexpected mechanism for amide bond inversion. It was found that the reaction does not proceed as direct N-C(O) bond inversion in the double-chair (CC) conformation but rather requires the conformational transformation into the chair–boat (CB) form first. The amide bond inversion in the latter requires less energy than in the CC form.

## 1. Introduction

**Scheme 1:**
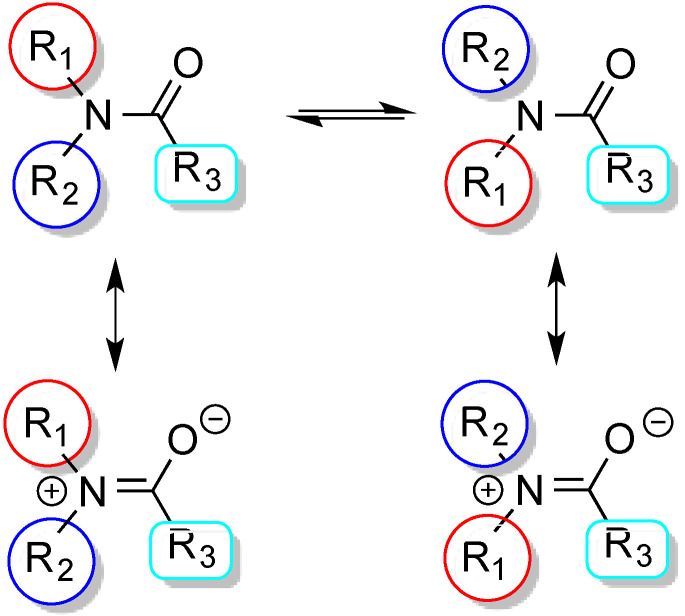
The general scheme of amide bond inversion.

Recently, we introduced the concept of “stereochemical chameleons”, i.e., functional groups which could exhibit either donor or acceptor behavior depending on their surroundings and conformation [1]. One of the most exciting examples of such groups is the amide group widespread in nature and extremely important synthetically. The typical and acknowledged property of the amide moiety is its planarity, which is a result of N-CO conjugation; in some cases, this planarity causes the existence of diastereomeric *E*- and *Z*-forms. Under some conditions, the amide group could undergo inversion of its configuration, which could be stable under other conditions [2,3] (Scheme 1).

Typical values for the rotational barriers around amide bonds range from 19.05 kcal/mol (R_1_ = R_2_ = R_3_ = Me, in water [4]) to 14.3 kcal/mol (R_1_ = R_2_ = Me, R_3_ = ferrocenyl (Fc), in CD_2_Cl_2_ [5]), being strongly dependent on all three R’s (Scheme 1) as well as on the nature of the solvent [6,7,8,9]. Szostak et al. established a useful correlation between the barrier of the amide bond rotation and the reactivity of amides in cross-coupling reactions [10].

**Scheme 2:**
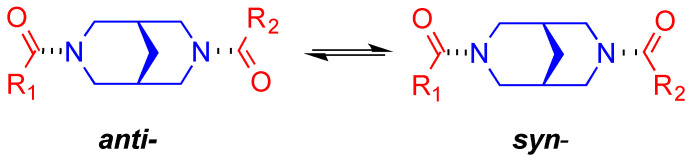
Two stereoisomeric forms that appear in the case of *N*,*N*′-diacylbispidines.

When one has two amide functionalities in one molecule, they could influence each other, provided they are spatially aligned in close proximity [2]. The bispidine (3,7-diazabicyclo[3.3.1]nonane) scaffold seems to be a highly useful platform in this respect since *N*,*N*′-bis-amides could exist in either *anti*- or *syn*-isomers [11] (Scheme 2).

The application of the *syn*/*anti* dichotomy in the chemistry of bispidines is well documented. For example, Palyulin et al. measured the ΔG of the *anti*/*anti* interconversion of 3,7-diacetylbispidine to be equal to 18.2 kcal/mol in DMF [12]. They also established the dependence of the *syn/anti* ratio on the polarity of the solvent (solvents such as CCl4, CDCl3, (CD3)2CO, (CD3)2SO, (CD3)2CO/CD3OD, CD3OD, CD3OD/D2O, and D2O were used). Wang et al. called the *anti*/*syn* interchange a “chiral–achiral switch” since, in the case R_1_ = R_2_ (see Scheme 2), the *anti*-form possesses C_2_ symmetry and thus should be chiral, whilst the *syn*-form has a symmetry plane and is therefore achiral [13]. They also pointed out that incorporation of 3,7-diacylbispidine in a medium-sized macrocycle could affect the total symmetry of the molecule.

The equilibrium constant for *anti*/*syn* interconversion strongly depends on the nature of the solvent: in more polar solvents, the *syn*-form predominates since it possesses a higher dipole moment compared to the *anti*-form [12,13,14]. It should be noted here that the presence of a metal ion in a solution could strongly affect the resulting *syn/anti* ratio towards the prevalence of the *syn*-form due to chelation of a metal center by two oxygen atoms, which are in close proximity in the *syn*-form [12,15].

At the same time, the *anti*-form would be more stable in less polar solvents and, presumably, in the crystal form since the crystallization of molecules with a smaller dipole moment is more preferable. A CSD search returns 19 structures of bispidine-based bis-amides (tricyclic compounds of the cytisine type are excluded) [16,17,18,19,20,21,22,23,24,25], out of which only 3 possess a *syn*-conformation in the crystal, and all belong to macrocyclic molecules containing two bispidine fragments [13,20,23].

*N*,*N*′-Diacyl bispidines have found various different applications in: medicinal chemistry [26] (a review), [27,28]; crystal engineering [29]; macroheterocycle synthesis [13,18,19,20,23,29,30,31]; secondary structure nucleators in peptides [22,32]; conformational switching [12,13]; lipid bilayer modifiers [15,33].

**Figure 1:**
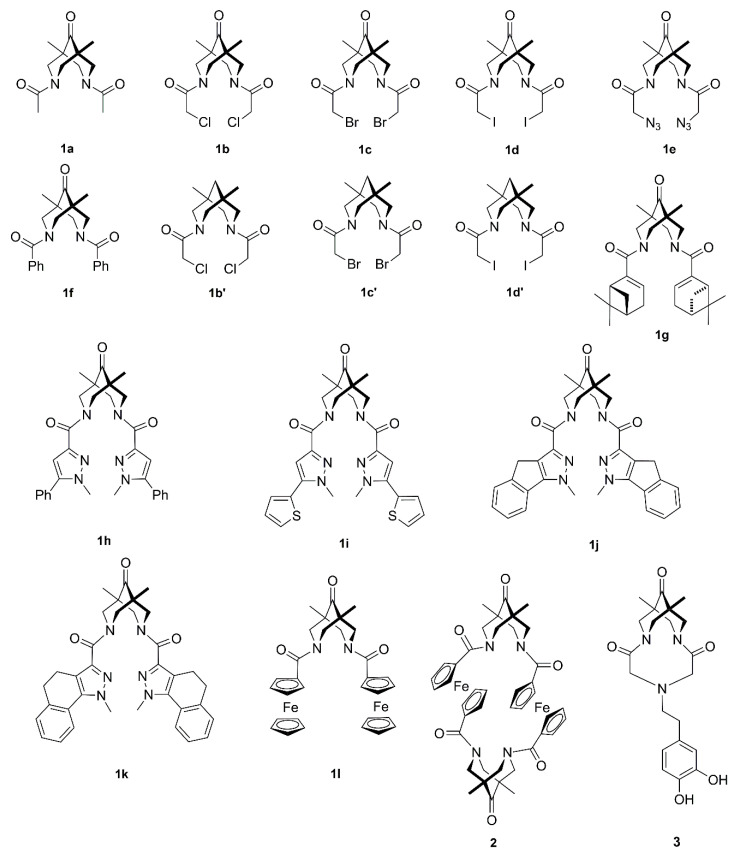
The structures and numbering of compounds discussed in this work.

In the frames of our studies on bispidine-type molecules as SARS-CoV-2 main protease inhibitors [27] and as components of catalytic systems for the Henry reaction [34], we focused our efforts on a group of symmetrical bispidine-based bis-amides. In this paper, we describe two crystal structures of new bispidinone bis-amides and an NMR study of the solution behavior of a group of bis-amides, **1**–**3** (Figure 1). The quantum chemical calculation of the amide bond rotation in bis-amide **1a** explaining the possible mechanism of the process is also presented.

## 2. Results and Discussion

### 2.1. Synthesis of Compounds

**Scheme 3:**
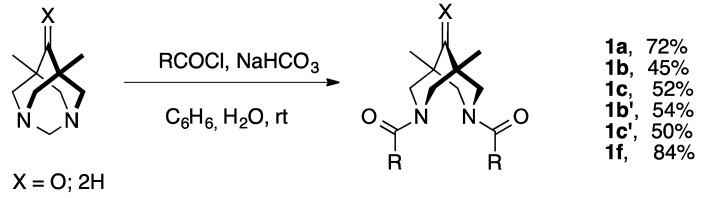
Synthesis of bis-amides by bridge splitting in 1,3-diazaadamantan-6-one and 1,3-diazaadamantane.

**Scheme 4:**
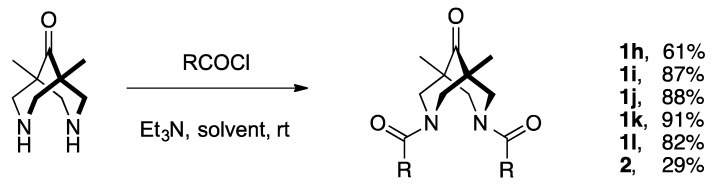
Synthesis of bis-amides by acylation of 1,5-dimethyl-3,7-diazabicyclo[3.3.1]nonan-9-one.

Bis-amides **1** and **2** were synthesized previously either from 5,7-dimethyl-1,3-diazaadamantan-6-one by methylene bridge cleavage in the biphasic system benzene–water (compounds **1a [12,35]** (for this compound, the acetic acid anhydride was used), **1b, 1c, 1b′**, **1c′ [25]**, and **1f [27]**, Scheme 3) or from 1,5-dimethyl-3,7-diazabicyclo[3.3.1]nonan-9-one by direct acylation with the corresponding acid chlorides (compounds **1h**–**1k [27]**, **1l,** and **2 [19]**, Scheme 4).

**Scheme 5:**
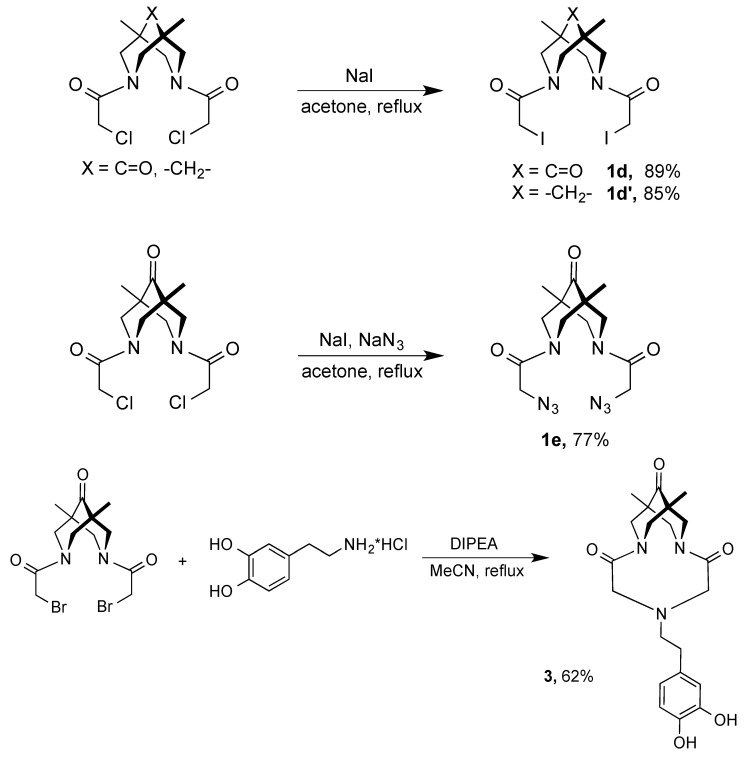
Synthesis of compounds **1d**, **1d′**, **1e**, and **3** by nucleophilic substitution reactions.

Amides **1d**, **1d′**, and **1e** were prepared by nucleophilic substitution reactions with sodium iodide and sodium azide in acetone, from the corresponding dichlorides (**1b** and **1b′**); the same approach was used for dopamine derivative **3** (Scheme 5; for experimental details, see Section 3 and Supplementary Materials, SM). The tricyclic core of molecules of type **3** is a well-known scaffold of AMPA receptor allosteric modulators [36].

**Scheme 6:**
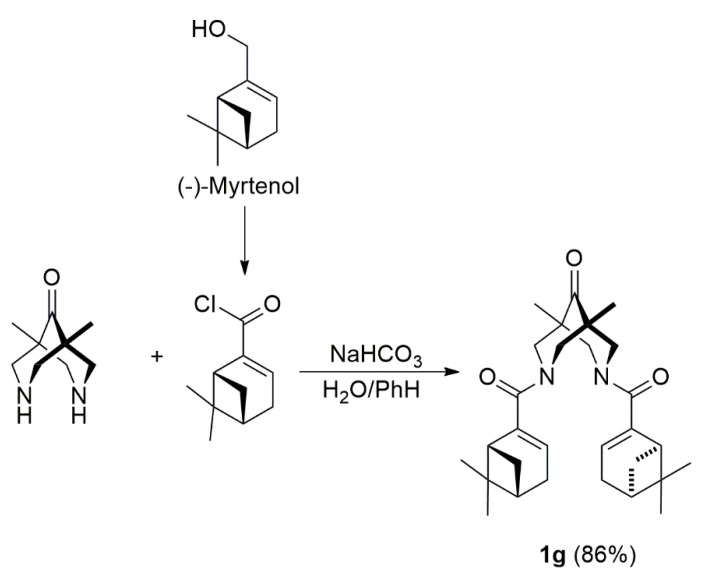
Synthesis of compound **1g**.

The chiral bis-amide **1g** was obtained by the interaction of the acid chloride of (1*R*)-(−)-myrtenic acid obtained from (-)-myrtenol with diazaadamantanone in a water–benzene mixture in the presence of NaHCO_3_ (Scheme 6). The effect of the compound on the physical activity of mice was recently studied [37]. Moreover, **1g** was used as a ligand of metal complex catalysts for the Henry reaction [34].

### 2.2. X-ray Studies

In this paper, we report two crystal structures, namely, those of compounds **1i** and **3**; in the latter, the relatively small macrocycle size (10 atoms) does not allow the formation of the *anti*-form; the solid-state conformation of the former is not as obvious.

**Figure 2:**
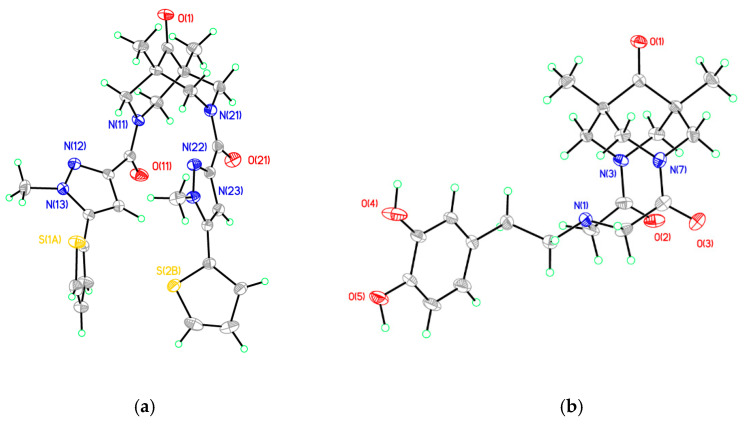
Molecular structures of **1i** (**a**) and **3** (**b**). Thermal ellipsoids are shown at a 50% probability level.

Surprisingly, molecules of **1i** adopt the *syn*-configuration in the crystal. In the structure of **1i**, the bispidinone skeleton adopts a chair–chair conformation with N…N separation equal to 2.869 Å (Figure 2a). Both amide fragments (CH_2_)_2_N-C(=O)-C are planar within 0.03 Å. The main geometrical parameters of the bicyclic core in the studied compounds are close to the values reported and discussed for several bispidine derivatives in [38] and references cited therein. The heterocycles in **1i** are close to an orthogonal disposition, and thus the observed conformation can be additionally stabilized by C-H…π interaction or S…π interaction.

We tentatively assumed that the solid-state *syn*-conformation of compound **1i** is due to attractive intramolecular interactions between two heterocyclic side arms. If this is the case, one would expect the existence of a high content of the *syn*-form in the solutions of the heterocyclic molecules with electron-rich aromatic fragments (compounds **1h**–**1k**), vide infra.

As it was suggested earlier, the 10-membered cycle in dopamine derivative **3** in the crystal exists in the *syn*-form. In the structure of **3**, the bispidinone skeleton exists in a chair–chair conformation with N…N separation equal to 2.776 Å (Figure 2b). Both amide fragments (CH_2_)_2_N-C(=O)-C are planar within 0.03 Å.

Of interest to mention is that the distances of O…O and C…C (O and C belong to the amide carbonyl groups) differ for both molecules: while in **1i**, O…O = 3.623 Å and C…C = 3.415 Å, in **3**, these values are 3.191 Å and 2.819 Å, respectively. These differences reflect the level of rigidity of the bispidine core: while the macrocyclic nature of **3** dictates the spatial proximity of the carbonyl groups in the 10-membered ring, the acyclic molecule **1i** could adopt a more relaxed conformation of the bicyclic core. The values of C…C separations found in published papers on bispidine bis-amides range from 3.041 [14] to 4.696 Å [13] (here, we compare only the C…C distance since O…O separation obviously differs for the *syn*- and *anti*-forms). These findings clearly demonstrate that, in contrast to the widely known conformational rigidity of the bispidine bicycle in the double-chair conformation, the subtle structural factors could have a pronounced impact on the spatial structure of the molecules, even in the case when a system contains conventionally planar and conformationally stable amide moieties. Indeed, the C(O)…C(O) distance in **3** seems to be the champion among other bispidine bis-amides.

### 2.3. NMR Studies

NMR studies were performed in two solvents, namely, CDCl_3_ and DMSO-*d*_6_, which differ in their polarity. This helped us to establish the basic characteristic of the conformational exchange between the *anti-* and *syn*-forms and to formulate some subtle conclusions. Please note that hereafter, we use the *syn*/*anti* ratio derived from the NMR spectra to estimate the influence of the substituent at the amide carbonyl on the conformational behavior of the molecule (see also SI for more information). The resulting data together with some literature data are shown in Table 1.

**Figure 3:**
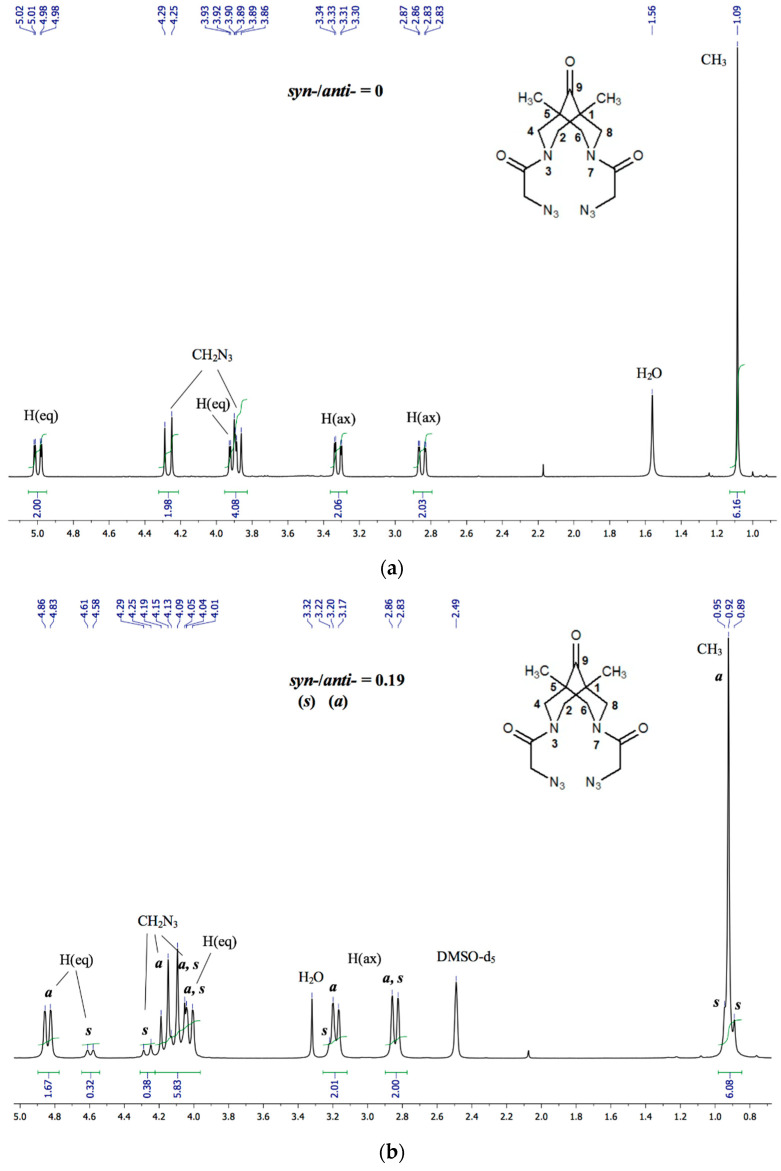
^1^H NMR spectrum of **1e** in CDCl_3_ (**a**); ^1^H-NMR spectrum of **1e** in DMSO-*d*_6_ (**b**).

In Figure 3, the ^1^H-NMR spectra of **1e** in CDCl_3_ (Figure 3a) and DMSO-d_6_ (Figure 3b) are shown as typical examples of the solvent polarity influence on the *syn*/*anti* ratio of bis-amides. These spectra demonstrate that while, in CDCl_3_, compound **1e** exhibits the presence of only the *anti*-form (the signals of methyl groups at positions 1 and 5 of the bicycle are equivalent; other signals prove the *C*_2_ symmetry), the solution in DMSO-*d*_6_ contains both *syn-* and *anti-*forms, as seen from the presence of two methyl signals, which is typical for a molecule possessing a symmetry plane passing through atoms 1, 9, and 5.

VT NMR experiments allowed us to estimate the rotation barriers of amide bond rotation for several molecules. The barriers of amide rotation for exchange between two positions with equal populations were estimated by measuring the coalescence temperatures for the chosen signals in the ^1^H-NMR and ^13^C spectra (see SI). Such experiments were performed for compounds **1i**, **1l**, and **1g** (for discussion of this particular compound, see below).

The assignment of the signals in the NMR spectra of **1g** in CDCl_3_ was carried out using 2D experiments (*COSY*, *NOESY*, *HSQC*, *HMBC*, see SI) and generally coincides with that in [37], with the exception of protons and carbon atoms of the bispidine framework (the assignment in that work was not fulfilled completely). We performed full assignment of all signals for **1g** in CDCl_3_ and DMSO-*d*_6_ in which VT experiments were carried out. The protons H^2,6^(eq) and H^4,8^(eq) were assigned taking into account their spatial closeness to the chiral substituent R.

It was found that compound **1g** exhibits more complex dynamic behavior compared to other bis-amides. At the slow exchange limit, the symmetry of the (*R*,*R*)-form (*anti*-isomer) should be *C_2_*, provided the substituents are free to rotate. At the fast exchange limit, the molecular symmetry is also, in principle, *C*_2_, but only due to two chiral substituents. However, their influence on the bispidine core is insignificant, meaning at high temperatures, its symmetry can be considered as pseudo-*C_2v_*.

The doubling of the H^2,6^(eq) signals in the ^1^H-NMR spectra, as well as the doubling of the C^3′^ and C^2′^ signals in the ^13^C-NMR spectra at room temperature, is apparently associated with the hindered rotation of bulky substituents with conjugated C=C–C=O double bonds around the N(CO)–C^2′^ bond (*C*_1_ symmetry). Thus, in this case, two processes are observed: amide rotation (Δ*G*^≠^ = 15.0 ± 0.3 kcal/mol) and rotation around the N(CO)–C^2′^ bond (Δ*G*^≠^ = 15.6 ± 0.3 kcal/mol).

**Table 1:** *Syn*/*anti* ratios for bis-amides **1a**–**1l**, **2**, and **3** in CDCl_3_ and DMSO-*d*_6_. Data are in ascending order of the *syn*-form in DMSO-*d*_6_.

#	Bis-amide	CDCl_3_	DMSO-*d*_6_	Data for X-ray Conformations and Solution Barriers
1.	**1g**	only *anti* *	only *anti* *	NMR: ΔG = 15.0 ± 0.3 kcal/mol (rotation around amide bond) * NMR: ΔG = 15.6 ± 0.3 kcal/mol (rotation around N(CO)–C^2′^ bond) *
2.	**2**	only *anti* [19]	only *anti* [19]	X-ray: bis-*anti* [19] NMR: no rotation around amide bond [19]
3.	**1f**	only *anti* *	only *anti* *	
4.	**1l**	only *anti* [19]	only *anti* [19]	X-ray: *anti* [19] NMR: ΔG = 14.5 ± 0.2 kcal/mol [19]
5.	**1e**	only *anti* *	0.19 *	
6.	**1a**	only *anti* *	0.21 *	X-ray: *anti* [21]
7.	**1b**	only *anti* [25]	0.25 [25]	
8.	**1c**	only *anti* [25]	0.33 [25]	X-ray: *anti* [25]
9.	**1b′**	only *anti* [25]	0.39 [25]	
10.	**1d**	only *anti* [25]	0.48 [25]	X-ray: *anti* [25]
11.	**1c′**	only *anti* [25]	0.54 [25]	X-ray: *anti* [25]
12.	**1d′**	only *anti* [25]	0.70 [25]	X-ray: *anti* [25]
13.	**1i**	0.33 [27]	1.00 *	X-ray: *syn* * NMR: ΔG = 16.3 ± 0.2 kcal/mol *
14.	**1h**	0.33 [27]	1.18 *	
15.	**1j**	1.43 [27]	2.33 *	
16.	**1k**	1.25 [27]	2.40 *	
17.	**3**	only *syn*	only *syn*	X-ray: *syn* *

* This work.

Although, at present, not all the data shown in Table 1 can be rationalized, we are able to comment on some results.

First of all, it is not easy to distinguish between pure electronic and steric reasons for the stability of a specific conformation. For example, the restricted rotation in the very tightly organized macrocycle **2** (line 2) dictates the resulting *anti*-form in both solvents. At the same time, in its acyclic analogue **1l** (line 4), in which rotation around the amide bond is possible, only the *anti-*form was found in both solvents. This finding, together with the results for the phenyl compound **1f** (line 3) and chiral terpene derivative **1g** (line 1), could reflect the steric repulsion between two bulky acyl substituents in a *syn*-form.

At the same time, the bulky pyrazole-containing molecules **1h**–**1k** (lines 13–16) also exhibit a high amount of the *syn*-form in both solvents. This might be tentatively explained by the possible intramolecular π–π interactions of the spatially proximal aromatic moieties in this form, which could stabilize it (see Figure 3a). The highest content of the *syn*-form for two homologous compounds, **1j** and **1k**, in which relatively planar and rigid tricyclic aromatic fragments are presented, additionally confirms this explanation.

The increase in the *syn*-form in acetic acid derivatives in the row **1e**–**1a**–**1b**–**1c**–**1d** presumably reflects the increase in the polarizability of the substituents, being lowest for H and N_3_ and highest for I (lines 5–8, 10). At the same time, the comparison of the *syn*/*anti* ratio between bispidin-9-ones **1b**–**d** and bispidines **1b′**–**1d′** shows that molecules with a methylene group in position 9 should possess bigger dipole moments than their carbonyl-containing counterparts. The reason for this is not clear, but we could explain it by the analysis of calculated dipole moments for **1a** and model compound **1a′** (**1a′** is 1,5-dimetyl-3,7-bis-acetyl-3,7-diazabicyclo[3.3.1]nonane, see below).

### 2.4. QC Calculations

To analyze the barriers and mechanism of the N-C(O) bond rotation of bispidines, we performed DFT modeling.

First of all, the geometry of **1a** was optimized using very tight optimization criteria and empirical dispersion corrections on the total energy with Becke–Johnson damping (D3). The minimum energy was observed for the CC conformation of the bicyclic skeleton and *anti*-arrangement of C=O groups, while for the *syn*-form, it was 4.7 kcal higher. At the same time, the *syn*-conformation of **1a** is characterized by a higher dipole moment (5.3 vs. 1.7 D, difference 3.6 D). Thus, one can assume that an increase in the solvent polarity would lead to a decrease in the energy difference for *syn* and *anti*. Indeed, the modeling of **1a** for the *syn-* and *anti-*arrangements of C=O groups using a solvent with a high dielectric constant leads to only a 1.3 kcal/mol difference. It should be noted that the same calculations for **1a′** (1,5-dimethyl-3,7-diacetyl-3,7-diazabicylo[3.3.1]nonane, the analogue of bisacetamide **1a** lacking a C=O group in the bridge position) lead to the same picture. Although the dipole moments for **1a′** are higher than for **1a** (7.0 and 5.0 D for the *syn*- and *anti*-forms, respectively), the difference in energy of isolated conformers as well as conformers with the account of solvent polarity is almost the same as in **1a** and equal to 4.8 and 1.2 kcal/mol. Thus, we can conclude that an increase in solvent polarity should lead to an increase in the *syn*-form in the case of **1a′** compared to **1a**, which is in qualitative agreement with the obtained experimental data (Table 1, lines 7 and 9, 8 and 11, 10 and 12). At the same time, it is clear that non-specific interactions, such as stacking in the case of heterocycles, hydrogen or halogen bonds, can significantly influence the equilibria.

The comparison of the molecular geometry for the *syn-* and *anti*-forms for **1a** showed that N…N contact in both of them practically coincides and is equal to 2.836 and 2.824 Å. The nature and energy of N…N interaction in **1a** were estimated using the topological analysis of the electron density distribution function ρ(r) within Bader’s quantum theory of “atoms in molecules” (QTAIM) [39]. Using the AIM formalism, one can distinguish the binding interatomic interactions from all other contacts. When the distribution of ρ(**r**) in the molecule or crystal is known, one can answer the question of whether the bonding interaction is present or not by searching the bond critical point (3,−1) and predicting the energy of weak intermolecular interactions (E_cont_) with high accuracy on the basis of the potential energy density function v(**r**)—the correlation suggested by Espinosa et al. (CEML) [40,41].

According to the critical point (CP) search of ρ(r), CP (3,−1), in **1a**, is found not only for all expected bonds but also for weak N…N interactions. It should be noted that for C…C and O…O contacts, the critical points (3,−1) were not located. Expectedly, all covalent bonds are characterized by both a negative ∇^2^ρ(r) and electron energy density (*h*_e_(*r*)) and thus correspond to the shared type of interatomic interactions, while N…N interactions are found to be of the closed shell type. The energies of N…N bonds according to CEML are equal to 3.7 kcal/mol.

**Figure 4:**
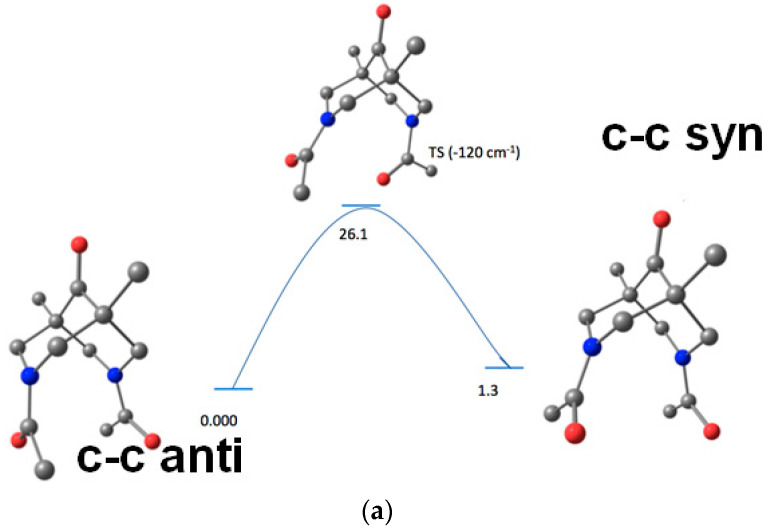

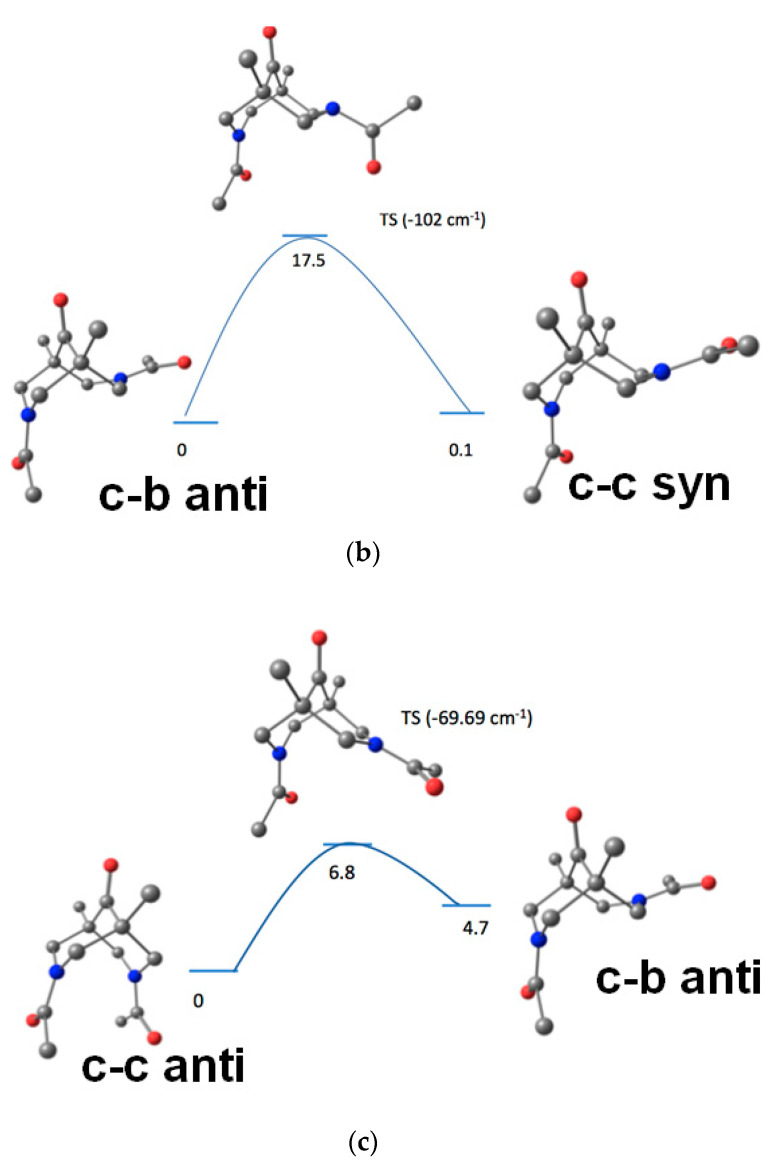
The calculated routes of *anti*/*syn* isomerization on the example of **1a**: direct amide inversion in the CC conformation (**a**); amide inversion in the CB conformation (**b**); CC–CB transformation (**c**).

Using the *syn-* and *anti*-forms as the starting geometry, we located the transition state for the barrier to rotation (Figure 4). As one can see, direct inversion of the amide bond costs too much (26.1 kcal/mol) and is probably unrealistic (Figure 4a). At the same time, if we propose that the exchange of an *anti*- to a *syn*-isomer occurs not in the chair–chair (CC) conformation, but in the chair–boat (CB) conformation, the barrier is lowered to 17.5 kcal/mol (Figure 4b). Of course, one should take into account that the difference in energy of the CC and CB conformations is 4.7 kcal/mol, and that the barrier of such a process is 6.8 kcal/mol (Figure 4c).

Thus, we can conclude that the more realistic mechanism for the transformation of the *anti*- to the *syn*-form includes the CC–CB conformational exchange of the bicyclic skeleton and consequent rotation around the amide bond.

## 3. Materials and Methods

All reagents and solvents used in the work (purity 90.0 – 99.9 + %) were purchased from commercial sources (SigmaAldrich, St. Louis, MO, USA; abcr, Karlsruhe, Dermany; Acros Organics, Fisher Scientific, Waltham, MA, USA), and, if necessary, subjected to further purification by standard routines immediately before use to achieve analytical purity. Solvents were purified by common methods. The reaction progress and purity of the obtained compounds were controlled by TLC on Merck Silicagel 60G F_254_ plates. Carl Roth Silica gel 60, 0.04–0.063 mm was used for column chromatography. High-resolution mass spectra with electrospray ionization were recorded on a Bruker MicroOTOF II instrument (Bruker AXS, Karlsruhe, Germany). Melting points were determined by the capillary method on Electrothermal IA9000 instrument (Cole-Parmer, Cambridgeshire, UK).

Compounds **1a** [42], **1b–d**, **1b′–1d′,** [25] **1l, 2,** [19] **1f**, **1h**–**1k [27]** were prepared as described earlier.

**Preparation of N,N′-bis(2-azidoacetyl)-1,5-dimethyl-3,7-diazabicyclo[3.3.1]nonan-9-one (1e).**

The solution of 0.73 mmol of N,N′-bis(2-chloroacetyl)-1,5-dimethyl-3,7-diazabicyclo[3.3.1]nonan-9-one (**1b**) in 12 mL of dry acetone was mixed with 2.2 mmol of sodium azide and 0.29 mmol of sodium iodide and refluxed for 16 h. The volatiles were removed, and the residue was suspended in water, filtered, and dried on air. The white residue was recrystallized form ethanol. Yield 77%. White fluffy needles. M.p. 199–201 °C (dec.), HRMS-ESI: Calc for [C_13_H_18_N_8_O_3_ + H]^+^ 335.1580. Found 335.1574.

NMR spectra see Figures S6–S8.

**Preparation of (3*r*,9*r*)-6-(3,4-dihydroxyphenethyl)-1,11-dimethyl-3,6,9-triazatricyclo[7.3.1.13,11]tetradecane-4,8,12-trione (3)**.

To the mixture of N,N′-bis(2-bromoacetyl)-1,5-dimethyl-3,7-diazabicyclo[3.3.1]nonan-9-one (**1c**, 755 mg, 1.86 mmol) and dopamine hydrochloride (352 mg, 1.86 mmol) in dry acetonitrile (22 ml) DIPEA (0.97 mL, 5.57 mmol) was added. The reaction mixture was refluxed with stirring for 6 h. The obtained solution was evaporated to dryness and extracted with DCM (250 mL) and water (15 mL). The organic fraction was separated, dried over sodium sulfate and evaporated to dryness. The product was purified with column chromatography on silica, eluent from DCM:MeOH 100:5 to DCM:MeOH 100:7.5. White powder. Yield 458 mg (62%). M.p. 267–269 °C (dec.). HRMS-ESI: Calc. for [C_21_H_27_N_3_O_5_ + H]^+^: 402.2023. Found: 402.2021.

X-Ray-quality crystals were obtained by slow evaporation of methanolic solution. 

NMR spectra see Figures S38–S40.

**X-ray structural determinations of 1i and 3.**

X-ray diffraction data were collected using Mo-*Kα* radiation (λ = 0.71073 Å) on a SMART APEX II area-detector diffractometer (Bruker AXS, Germany; graphite monochromator, *ω*-scan technique). The intensity data were integrated by the SAINT program [43] and corrected for absorption and decay by SADABS [44]. All structures were solved by direct methods using SHELXT [45] and refined against *F^2^* using SHELXL-2018 [46]. Analysis of anisotropic displacement parameters has revealed that in **1i** thiophen molecules are disordered by two positions leading to the superposition of sulfur and C-H. The refinement of two different positions for each of the fragments was done refined with the EADP and DFIX constraints. In addition to the disorder of the complex in the crystals, disorder is observed for lattice solvate molecule which in the case of **3** was removed by the SQUEEZE method [47] (implemented in the PLATON program) [48]. All C-H hydrogen atoms were placed in ideal calculated positions and refined as riding atoms with relative isotropic displacement parameters. The SHELXTL program suite [43] was used for molecular graphics. Crystal data, data collection, and structure refinement details are summarized in Table S1.

**NMR study**

^1^H and ^13^C-NMR spectra were recorded at room temperature on Bruker Avance-400, Bruker Avance-III-500 and Bruker Avance-600 spectrometers (Bruker AXS, Germany). The spectrometer frequency is denoted in parentheses for each spectral data set. Chemical shifts were referred to the signals of the deuterated solvents (7.26 ppm and 77.0 ppm for CDCl_3_, 2.49 ppm and 39.5 ppm for (CD_3_)_2_SO). The variable temperature experiments were performed on a Bruker Avance-III-500 spectrometer (Bruker AXS, Germany). When necessary, the assignment of signals in the NMR spectra was carried out using 2D techniques. Lorentz-Gauss apodization was used for precise measurement of the proton coupling constants values.

The barriers of amide rotation for exchange between two positions with equal populations were estimated by measuring the coalescence temperatures for the chosen signals in the ^1^H-NMR spectra using Equation (1):

*k*_coal_ = (*kT*_coal_/*h*)·exp(−Δ*G*^≠^/*RT*_coal_) = π(δν)/√2 (1)

where δν is the difference (in Hz) between two signals at the slow exchange limit.

Compound **1g** (*syn*-/*anti*- = 0).

^1^H-NMR (500 MHz, (CD_3_)_2_SO):

*T*_coal_(NCH_2_(ax)) ≈ *T*_coal_(NCH_2_(eq)) ≈ *T*_coal_(H^2,6^(eq)) ≈ 323 K (50 °C)

δν(NCH_2_(ax)) = δν(NCH_2_(eq)) = 220 Hz (298 K (25 °C)); Δ*G*^≠^ = 62.7 ± 1.2 kJ/mol (15.0 ± 0.3 kcal/mol) (**amide rotation**)).

δν(H^2,6^(eq)) = 81 Hz (298 K (25 °C)); Δ*G*^≠^ = 65.4 ± 1.2 kJ/mol (15.6 ± 0.3 kcal/mol) (**rotation around N(CO)–C^2′^ bond**).

The temperature of the slow exchange limit for ^1^H spectra is slightly lower than the ambient temperature (the lines are still broad at 298 K), so the accuracy of the Δ*G*^≠^ estimate for ^1^H spectra is lower than for **1i** and **1l** (see below). 

^13^C-NMR (500 MHz, (CD_3_)_2_SO):

*T*_coal_(NCH_2_) ≈ 333 K (60 °C)

δν(NCH_2_) = 594 Hz (298 K (25 °C)); Δ*G*^≠^ = 61.9 ± 1.2 kJ/mol (14.8 ± 0.3 kcal/mol) (**amide rotation**)).

Compound **1i** (*syn*-/*anti*- = 1.00). ^1^H-NMR (500 MHz, (CD_3_)_2_SO):

*T*_coal_(NCH_3_) = 341 K (68 °C); δν = 107.4 Hz (298 K (25 °C)); Δ*G*^≠^ = 68.3 ± 0.9 kJ/mol (16.3 ± 0.2 kcal/mol).

*T*_coal_(H^4′^) = 347 K (74 °C); δν = 165.3 Hz (298 K (25 °C)); Δ*G*^≠^ = 68.4 ± 0.9 kJ/mol (16.3 ± 0.2 kcal/mol).

Compound **1l** (*syn*-/*anti*- = 0). ^1^H-NMR (500 MHz, CDCl_3_):

*T*_coal_(NCH_2_(ax)) = 306 K (33 °C); δν = 118.2 Hz (255 K (−18 °C)); Δ*G*^≠^ = 60.8 ± 0.9 kJ/mol (14.5 ± 0.2 kcal/mol).

## 4. Conclusions

In this work, we demonstrated that the *anti*/*syn* ratio of bispidine bis-amides could be affected not only by changing the solvent polarity, but also by the nature of the acylic substituents. Bispidines with aliphatic and heterocyclic units in the acylic R(CO) fragments were found to possess an increased content of the *syn*-form in DMSO-d_6_ solutions. In the case when R = Ph, ferrocenyl, (*R*)-myrtenyl, only the *anti*-form was detected both in CDCl_3_ and DMSO-*d*_6_.

For the first time, VT NMR experiments were applied to a compound derived from a natural terpene, myrtene, and a new dynamic process was found in addition to the expected inversion around the amide N-C(O) bond.

Quantum chemical calculations revealed that the reaction of amide bond inversion proceeds via the conformational transformation into the chair–boat (CB) form, with subsequent inversion of this form taking place.

The results of this work could find application in mechanistic studies of the biological action of bispidine-based bis-amides and in the design of new biologically active molecules. For example, according to docking studies, only one isomeric form of compound **1k** was found to be covalently bound to the active site of the SARS-CoV-II main protease [29].

## Data Availability

Not applicable.

## Supplementary Materials

The following Supporting Information can be downloaded online, Table S1. Crystal data, data collection, and structure refinement details for **1i** and **3**; Figures S1–S40—NMR data; Details of quantum chemistry calculations.

## Sample Availability

Not available.
