# Supplementary material for: Bispidine Platform as a Tool for Studying Amide Configuration Stability
Source: Molecules. 2022 Jan 10;27(2):430. doi: 10.3390/molecules27020430 (PMC8779339; doi:10.3390/molecules27020430)
Supplement: Supplementary file 1 [file molecules-27-00430-s001.zip › molecules-1518090-supplementary.pdf]

Supporting Materials

Bispidine Platform as a Tool for Studying Amide Configuration Stability

Dmitry P. Krut'ko,[1] Aleksei V. Medved'ko,[2] Konstantin A. Lyssenko,[1] Andrei V. Churakov,[3] Alexander I. Dalinger,[1] Mikhail A. Kalinin,[1,2] Alexey O. Gudovannyy,[1,4] Konstantin Y. Ponomarev,[5] Eugeny V. Suslov,[5] Sergey Z. Vatsadze[1] *

[1] *Chemistry Department, Lomonosov Moscow State University, Leninskie Gory, MSU, 1-3, 119991 Moscow, Russian Federation*
[2] *Zelinsky Institute of Organic Chemistry, RAS, Leninsky Pr., 47, 119991 Moscow, Russian Federation*
[3] *N.S. Kurnakov Institute of General and Inorganic Chemistry, RAS, Leninsky Pr., 31, 119991 Moscow, Russian Federation*
[4] *Mendeleev University of Chemical Technology of Russia, Miusskaya square, 9, 125047 Moscow, Russian Federation*
[5] *N.N. Vorozhtsov Novosibirsk Institute of Organic Chemistry, SB RAS, acad. Lavrentjev ave., 9, 630090 Novosibirsk, Russia*
e-mail: zurabych@gmail.com

Table S1. Crystal data, data collection, and structure refinement details for **1i** and **3.** 2
NMR study 3
Compound **1a** 3
Compound **1e** 6
Compound **1f** 9
Compound **1g** 10
Compound **1h** 20
Compound **1i** 21
Compound **1j** 23
Compound **1k** 26
Compound **3** 29
Quantum chemistry calculations 32
References 33

Table S1. Crystal data, data collection, and structure refinement details for **1i** and **3**.

	**1i**	**3**
Empirical formula	$C_{27}H_{28}N_6O_3S_2$	$C_{23}H_{33}N_3O_6$
Formula weight	548.67	447.52
Temperature (K)	150	120
Crystal system	Monoclinic	Monoclinic
Space group	$P2_1/n$	$P2_1/c$
Z(Z’)	4(1)	4(1)
a (Å)	9.4919(11)	21.4608(18)
b (Å)	26.726(3)	7.3606(6)
c (Å)	10.2141(12)	13.2599(10)
α (°)	90.00	90
β (°)	98.090(2)	91.094(2)
γ (°)	90.00	90
Volume (Å^3)	2565.3(5)	2094.2(3)
ρ_{calc}, g/cm^3	1.421	1.419
m, cm^{-1}	2.51	1.03
F(000)	1152	960
$2q_{max}$, °	58	50
Refl. collected	28267	13414
Refl. unique (R_{int})	6811	5497
Refl. with I > 2σ(I)	5598	3982
Parameters	349	285
R_1 with I > 2σ(I)	0.0412	0.0628
wR_2 (all data)	0.1099	0.1796
Goodness-of-fit on F^2	1.038	0.986
Largest difference in peak/hole (e/Å^3)	0.422/-0.618	0.302/-0.297
CCDC	1860310	2126246

NMR Study

NMR spectra of **1b** – **1d**, **1b'** -**1d'** in $CDCl_3$ and $(CD_3)_2SO$ see [1]; **1l**, **2** in $CDCl_3$ and $(CD_3)_2SO$ see [2]; **1f**, **1h** - **1k** in $CDCl_3$ see [3].

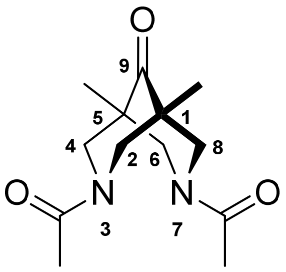

1a

Compound **1a** (*anti*-). ^{1}H NMR (600 MHz, $CDCl_3$, δ/ppm, *J*/Hz): 1.03 (s, 6 H, $C^{1,5}CH_3$); 2.15 (s, 6 H, $C(O)CH_3$); 2.75 (dd, 2 H, $^2J_{HH}$ = 13.8, $^4J_{HH}$ = 2.5, $H^{2,6}$(ax) or $H^{4,8}$(ax)); 3.28 (dd, 2 H, $^2J_{HH}$ = 13.4, $^4J_{HH}$ = 2.5, $H^{4,8}$(ax) or $H^{2,6}$(ax)); 4.07 (dd, 2 H, $^2J_{HH}$ = 13.4, $^4J_{HH}$ = 2.8, $H^{4,8}$(eq) or $H^{2,6}$(eq)); 5.04 (dd, 2 H, $^2J_{HH}$ = 13.8, $^4J_{HH}$ = 2.8, $H^{2,6}$(eq) or $H^{4,8}$(eq)). ^{13}C NMR (100 MHz, $CDCl_3$, δ/ppm): 16.50 ($C^{1,5}$*C*H_3); 21.47 (C(O)*C*H_3); 45.86 ($C^{1,5}$); 53.16, 57.77 (CH_2N); 169.96 (C(O)N); 211.67 (C^9=O).

Compound **1a** (*syn*-/*anti*- = 0.21). ^{1}H NMR (400 MHz, $(CD_3)_2SO$, δ/ppm, *J*/Hz): 0.90, 0.91 (both s, $C^{1,5}CH_3$ (*syn*-)); 0.91 (s, $C^{1,5}CH_3$ (*anti*-)); 2.00 (s, $C(O)CH_3$ (*anti*-)); 2.07 (s, $C(O)CH_3$ (*syn*-)); 2.69 (br d, $^2J_{HH}$ = 13.3, $H^{2,8}$(ax) or $H^{4,6}$(ax) (*syn*-)); 2.73 (dd, $^2J_{HH}$ = 13.4, $^4J_{HH}$ = 2.6, $H^{2,6}$(ax) or $H^{4,8}$(ax) (*anti*-)); 3.23 (dd, $^2J_{HH}$ = 13.4, $^4J_{HH}$ = 2.6, $H^{4,8}$(ax) or $H^{2,6}$(ax) (*anti*-)); 3.24 (br d, $^2J_{HH}$ = 13.3, $H^{4,6}$(ax) or $H^{2,8}$(ax) (*syn*-)); 4.11(dd, $^2J_{HH}$ = 13.4, $^4J_{HH}$ = 2.9, $H^{4,8}$(eq) or $H^{2,6}$(eq) (*anti*-)); 4.30 (br d, $^2J_{HH}$ = 13.3, $H^{4,6}$(eq) or $H^{2,8}$(eq) (*syn*-)); 4.59 (br d, $^2J_{HH}$ = 13.3, $H^{2,8}$(eq) or $H^{4,6}$(eq) (*syn*-)); 4.85 (dd, $^2J_{HH}$ = 13.4, $^4J_{HH}$ = 2.9, $H^{2,6}$(eq) or $H^{4,8}$(eq) (*anti*-)). ^{13}C NMR (100 MHz, $(CD_3)_2SO$, δ/ppm): 15.98, 16.61 ($C^{1,5}$*C*H_3 (*syn*-)); 16.05 ($C^{1,5}$*C*H_3 (*anti*-)); 21.33 (C(O)*C*H_3 (*syn*-)); 21.43 (C(O)*C*H_3 (*anti*-)); 45.07 ($C^{1,5}$ (*syn*-)); 45.34 ($C^{1,5}$ (*syn*-, *anti*-)); 52.32, 56.87 (CH_2N (*anti*-)); 53.10, 56.52 (CH_2N (*syn*-)); 168.24 (C(O)N (*syn*-)); 168.85 (C(O)N (*anti*-)); 212.25 (C^9=O (*anti*-)).

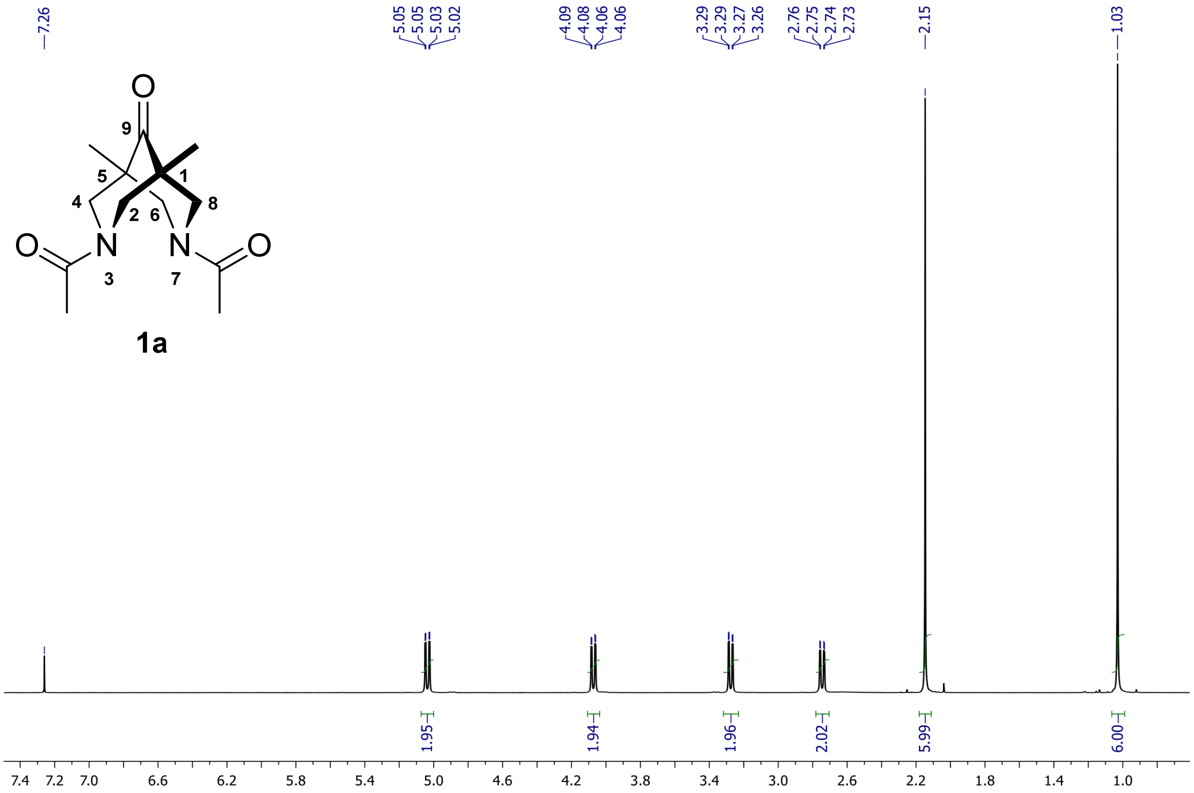

Figure S1. ^{1}H NMR spectrum of **1a** in $CDCl_3$.

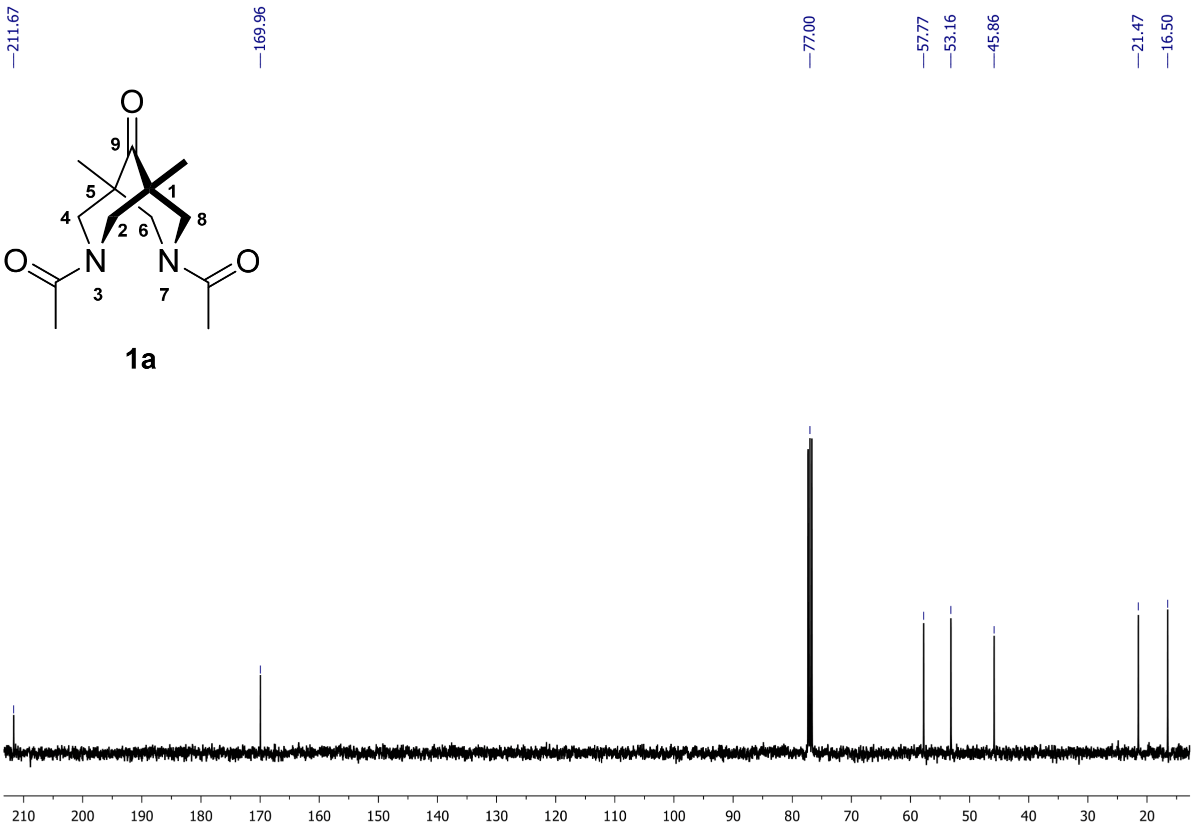

Figure S2. ^{13}C NMR spectrum of **1a** in $CDCl_3$.

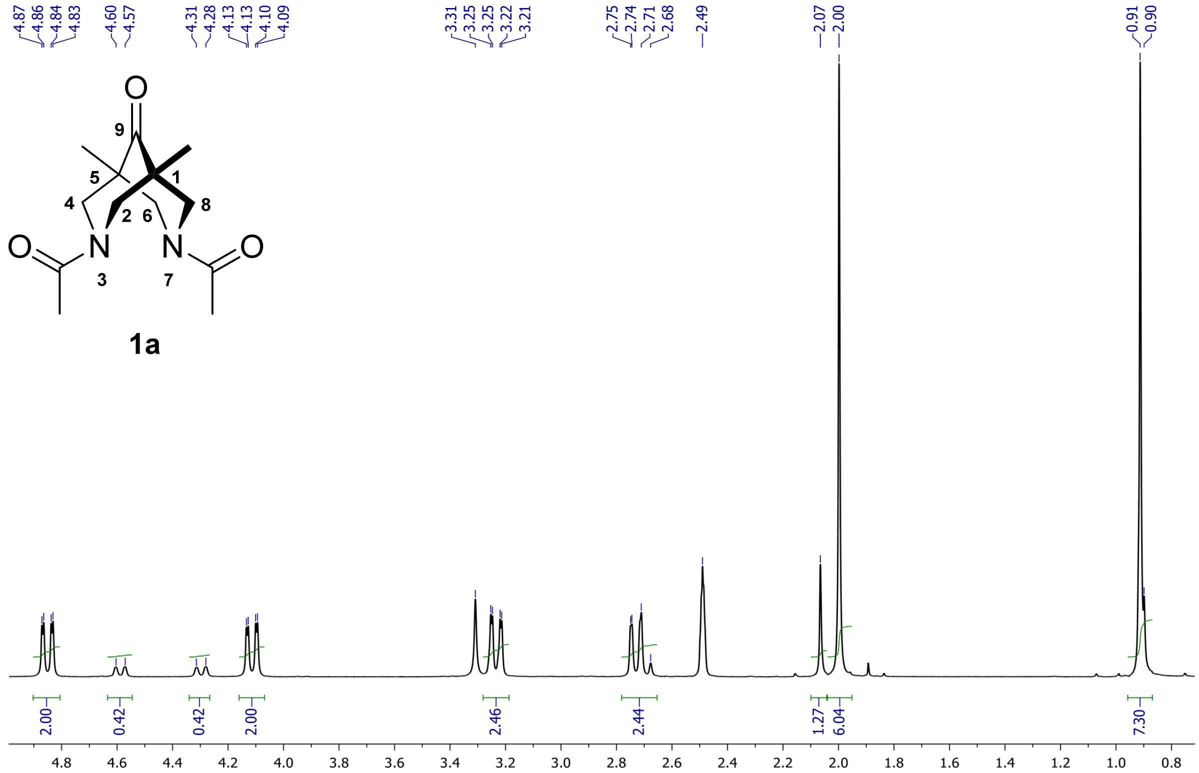

Figure S3. ^{1}H NMR spectrum of **1a** in DMSO-d6.

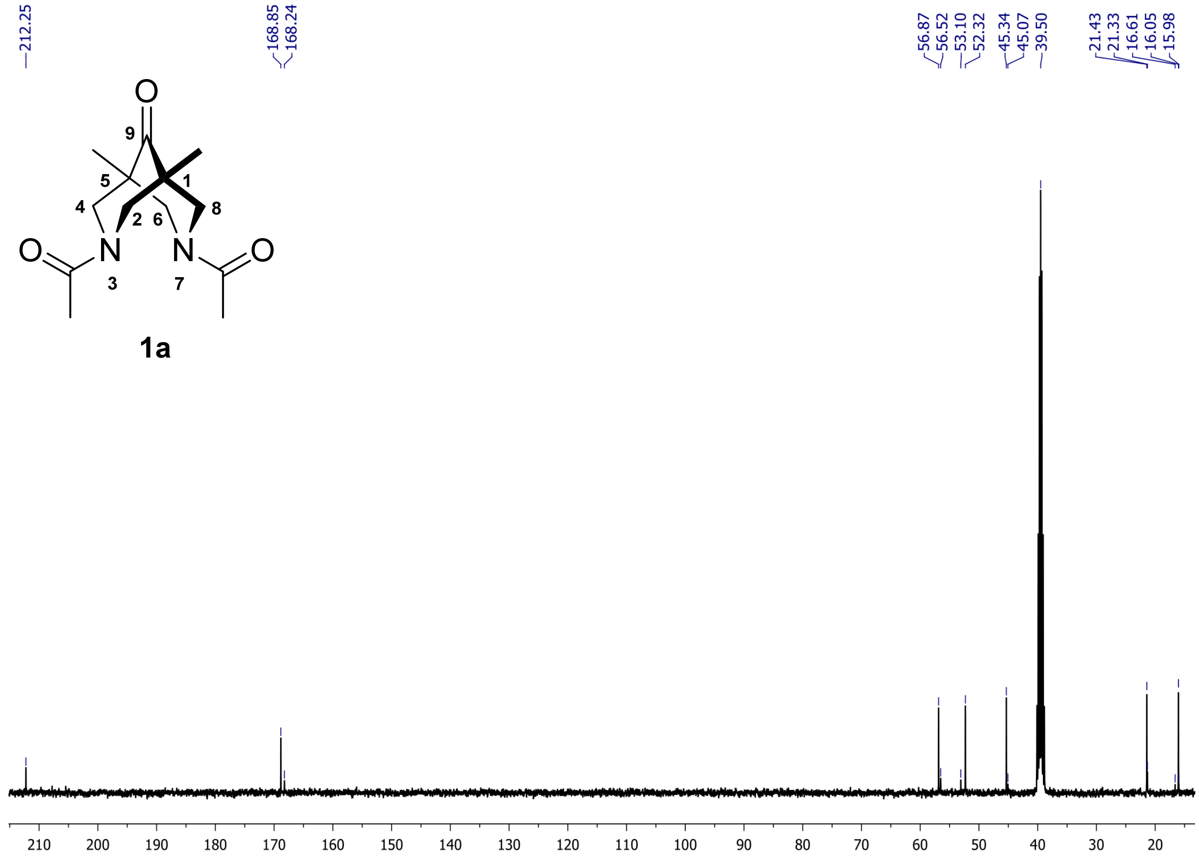

Figure S4. ^{13}C NMR spectrum of **1a** in DMSO-d6.

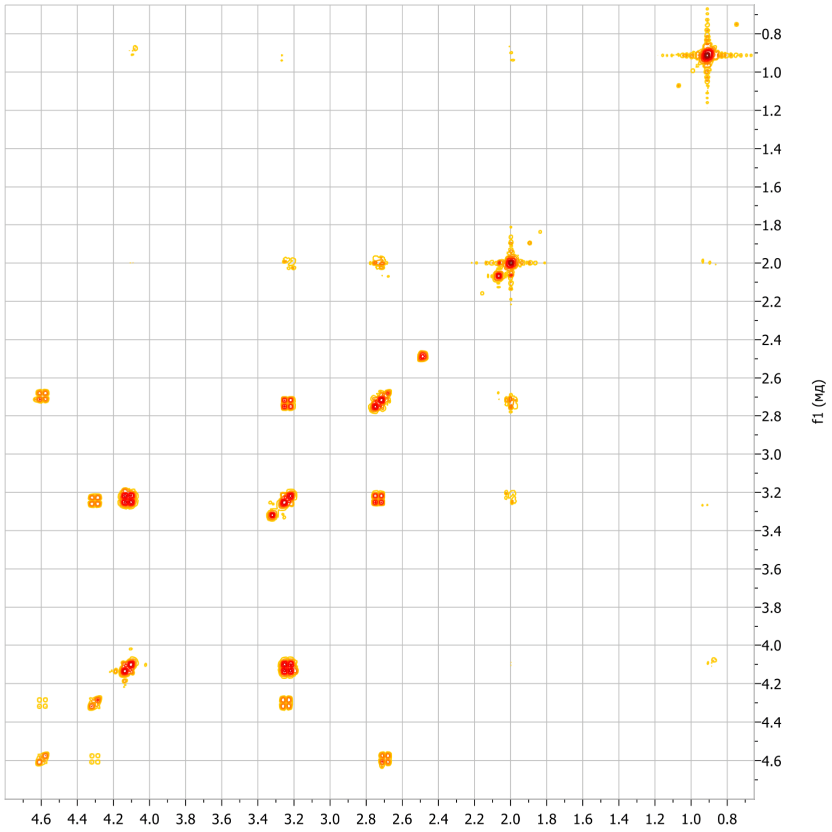

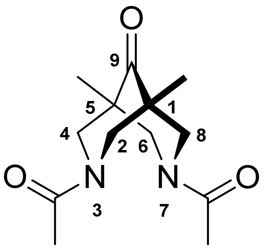

1a

Figure S5. *COSY* NMR spectrum of **1a** in DMSO-d$_6$.

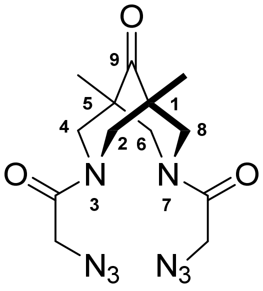

1e

Compound **1e** (*anti*-). ^{1}H NMR (400 MHz, CDCl$_3$, δ/ppm, *J*/Hz): 1.09 (s, 6 H, CH$_3$); 2.84 (dd, 2 H, $^2J_{HH}$ = 14.0, $^4J_{HH}$ = 2.5, H^{2,6}(ax) or H^{4,8}(ax)); 3.32 (dd, 2 H, $^2J_{HH}$ = 13.5, $^4J_{HH}$ = 2.5, H^{4,8}(ax) or H^{2,6}(ax)); 3.88, 4.27 (both d, 2 H, $^2J_{HH}$ = 15.6, CH$_2$N$_3$); 3.91 (dd, 2 H, $^2J_{HH}$ = 13.5, $^4J_{HH}$ = 2.9, H^{4,8}(eq) or H^{2,6}(eq)); 5.00 (dd, 2 H, $^2J_{HH}$ = 14.0, $^4J_{HH}$ = 2.9, H^{2,6}(eq) or H^{4,8}(eq)).

Compound **1e** (*syn*-/*anti*- = 0.19). ^{1}H NMR (400 MHz, (CD$_3$)$_2$SO, δ/ppm, *J*/Hz): 0.89, 0.95 (both s, CH$_3$ (*syn*-)); 0.92 (s, CH$_3$ (*anti*-)); 2.84 (d, $^2J_{HH}$ = 13.4, H^{2,6}(ax) or H^{4,8}(ax) (*anti*-), H^{2,8}(ax) or H^{4,6}(ax) (*syn*-)); 3.18 (d, $^2J_{HH}$ = 13.6, H^{4,8}(ax) or H^{2,6}(ax) (*anti*-)); 3.21 (d, $^2J_{HH}$ = 13.2, H^{4,6}(ax) or H^{2,8}(ax) (*syn*-)); 4.02 (d, $^2J_{HH}$ = 13.6, H^{4,8}(eq) or H^{2,6}(eq) (*anti*-)); 4.08, 4.16 (*AB*-system, $^2J_{HH}$ = 16.5, CH$_2$N$_3$ (*anti*-)); 4.27 (lowfield part of *AB*-system, $^2J_{HH}$ = 16.6, CH$_2$N$_3$ (*syn*-)); highfield part of *AB*-system and the doublet of H^{4,6}(eq) or H^{2,8}(eq) (*syn*-) are obscured by the signals of *anti*-isomer in the range 4.0-4.2 ppm; 4.60 (d, $^2J_{HH}$ = 13.3, H^{2,8}(eq) or H^{4,6}(eq)

(*syn*-)); 4.84 (d, $^{2}J_{HH}$ = 13.4, $H^{2,6}$(eq) or $H^{4,8}$(eq) (*anti*-)). ^{13}C NMR (100 MHz, $(CD_3)_2SO$, δ/ppm): 15.78 (CH_3 (*anti*-)); 15.90, 16.44 (CH_3 (*syn*-)); 44.98, 45.39 ($C^{1,5}$ (*syn*-)); 45.57 ($C^{1,5}$ (*anti*-)); 49.42 (CH_2N_3 (*syn*-)); 49.88 (CH_2N_3 (*anti*-)); 52.57, 54.82 (CH_2N (*anti*-)); 53.63, 54.48 (CH_2N (*syn*-)); 166.14 (C(O)N (*syn*-)); 166.62 (C(O)N (*anti*-)); 211.32 (C^9=O (*anti*-)).

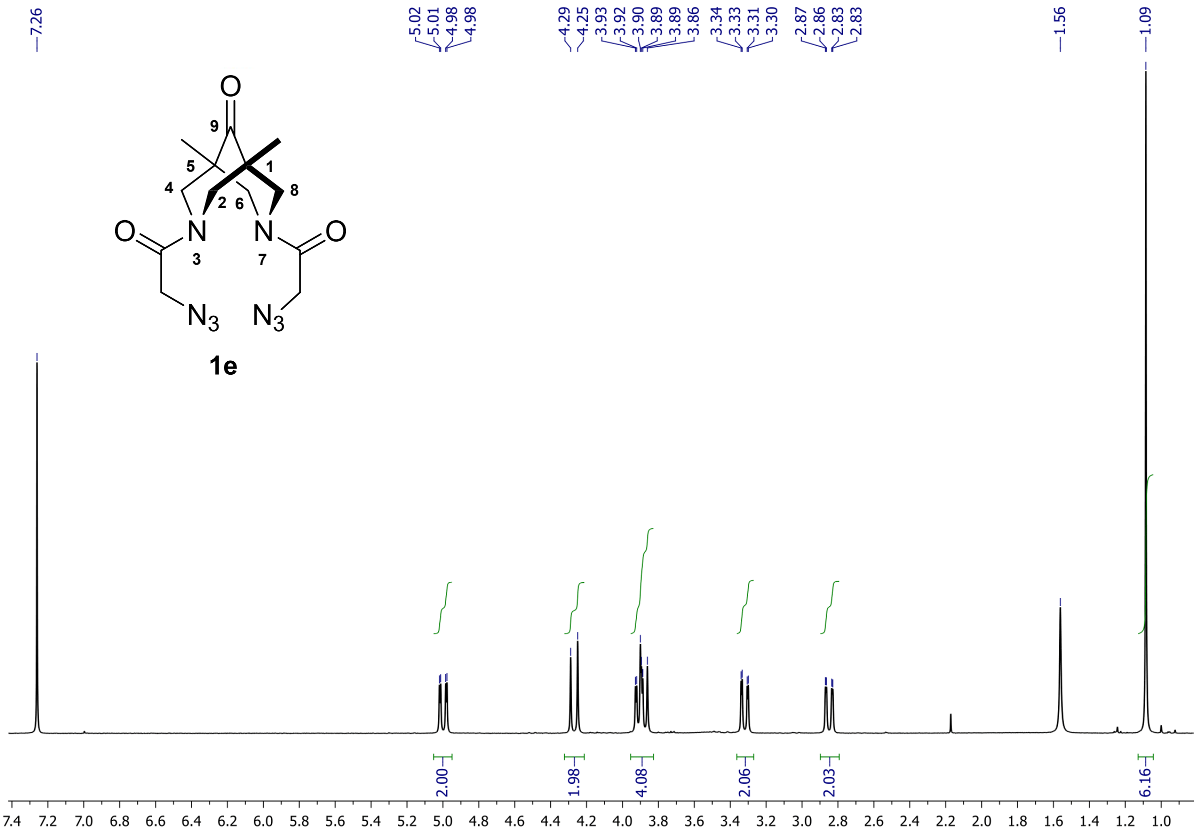

Figure S6. ^{1}H NMR spectrum of **1e** in $CDCl_3$.

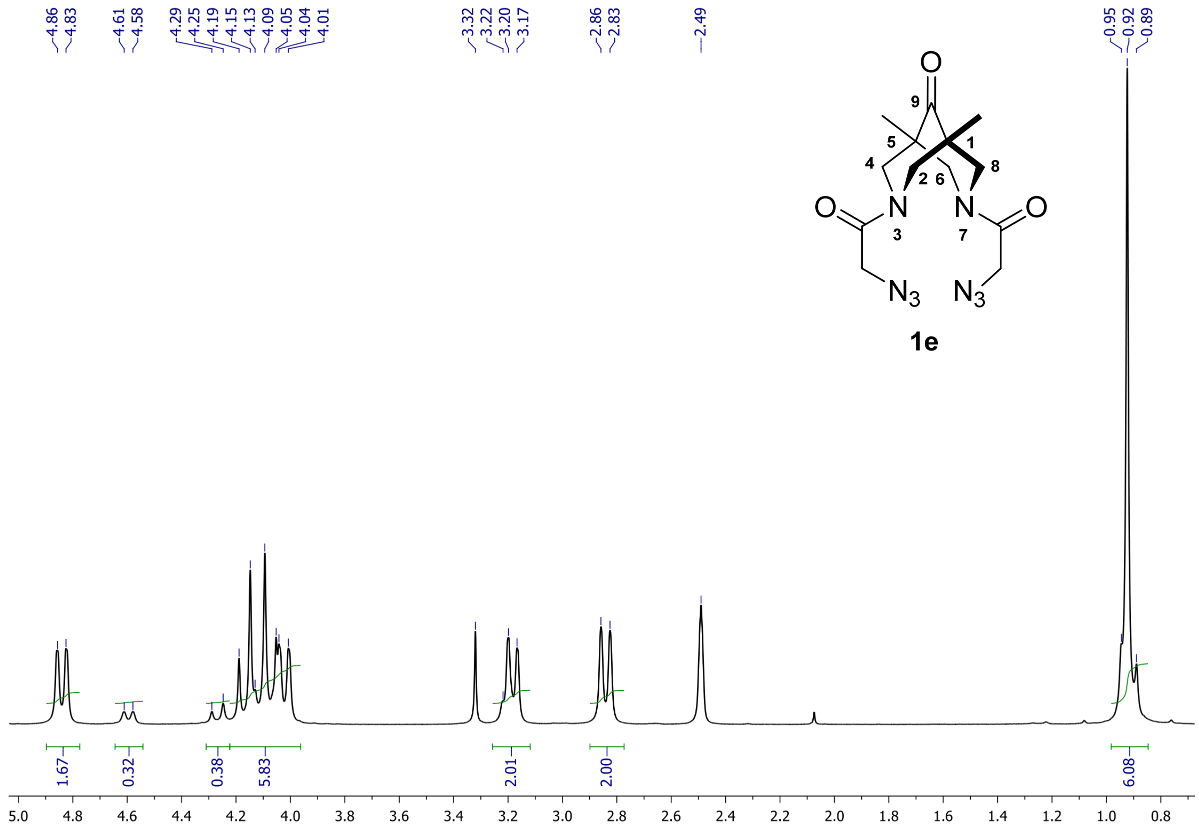

Figure S7. ^{1}H NMR spectrum of **1e** in DMSO-d6.

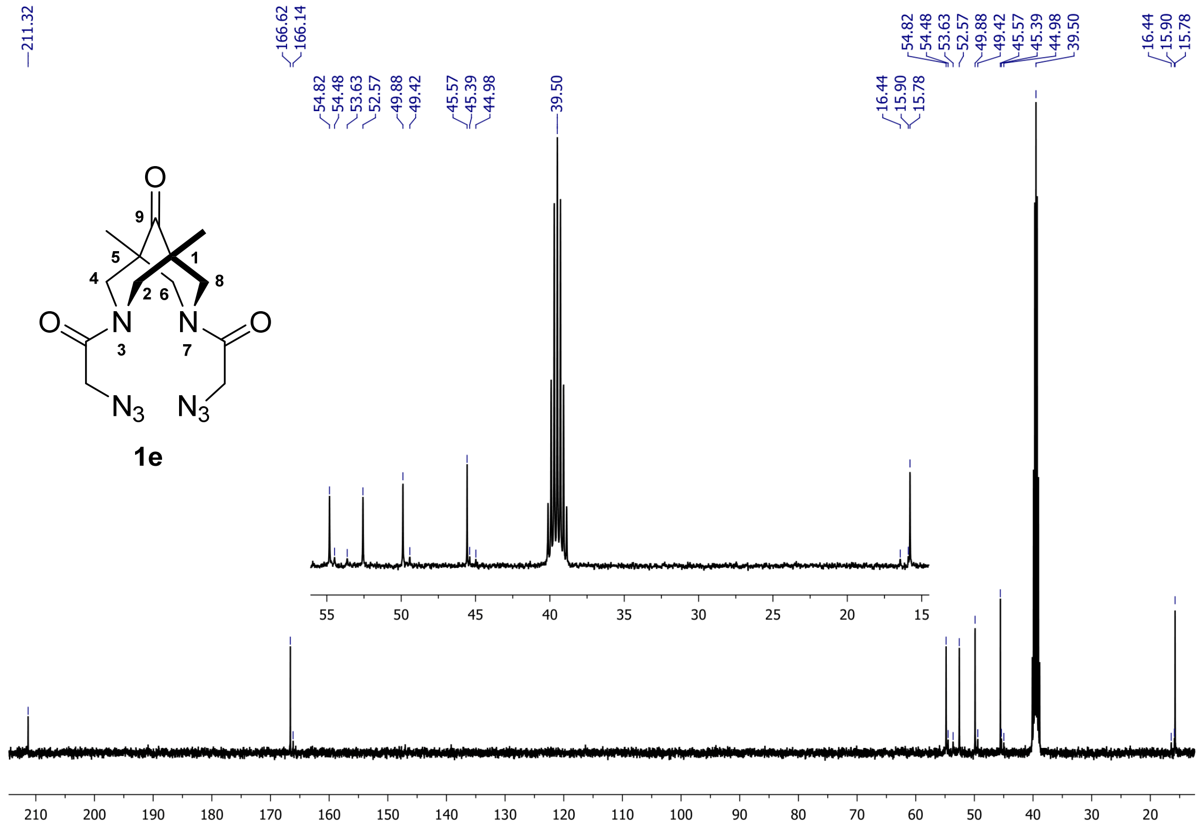

Figure S8. ^{13}C NMR spectrum of **1e** in DMSO-d6.

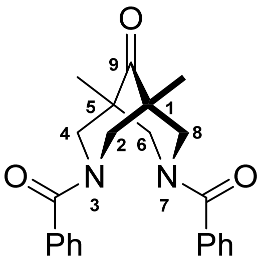

1f

Compound **1f** (*anti*-). ^{1}H NMR (400 MHz, $(CD_3)_2SO$, δ/ppm, *J*/Hz): 0.86 (s, 6 H, CH_3); 3.02 (d, 2 H, $^{2}J_{HH}$ = 13.5, $H^{2,6}$(ax) or $H^{4,8}$(ax)); 3.49 (d, 2 H, $^{2}J_{HH}$ = 12.9, $H^{4,8}$(ax) or $H^{2,6}$(ax)); 3.84 (d, 2 H, $^{2}J_{HH}$ = 12.9, $H^{4,8}$(eq) or $H^{2,6}$(eq)); 4.70 (d, 2 H, $^{2}J_{HH}$ = 13.5, $H^{2,6}$(eq) or $H^{4,8}$(eq)); 7.45 (m, 6 H, *p*-H, *m*-H); 7.50 (m, 4 H, *o*-H). ^{13}C NMR (100 MHz, $(CD_3)_2SO$, δ/ppm): 16.25 (CH_3); 45.59 ($C^{1,5}$); 53.12, 58.76 (CH_2N); 127.49, 128.22 (*o*-CH, *m*-CH); 129.55 (*p*-CH); 135.69 (*i*-C); 169.49 (C(O)N); 212.09 (C^9=O).

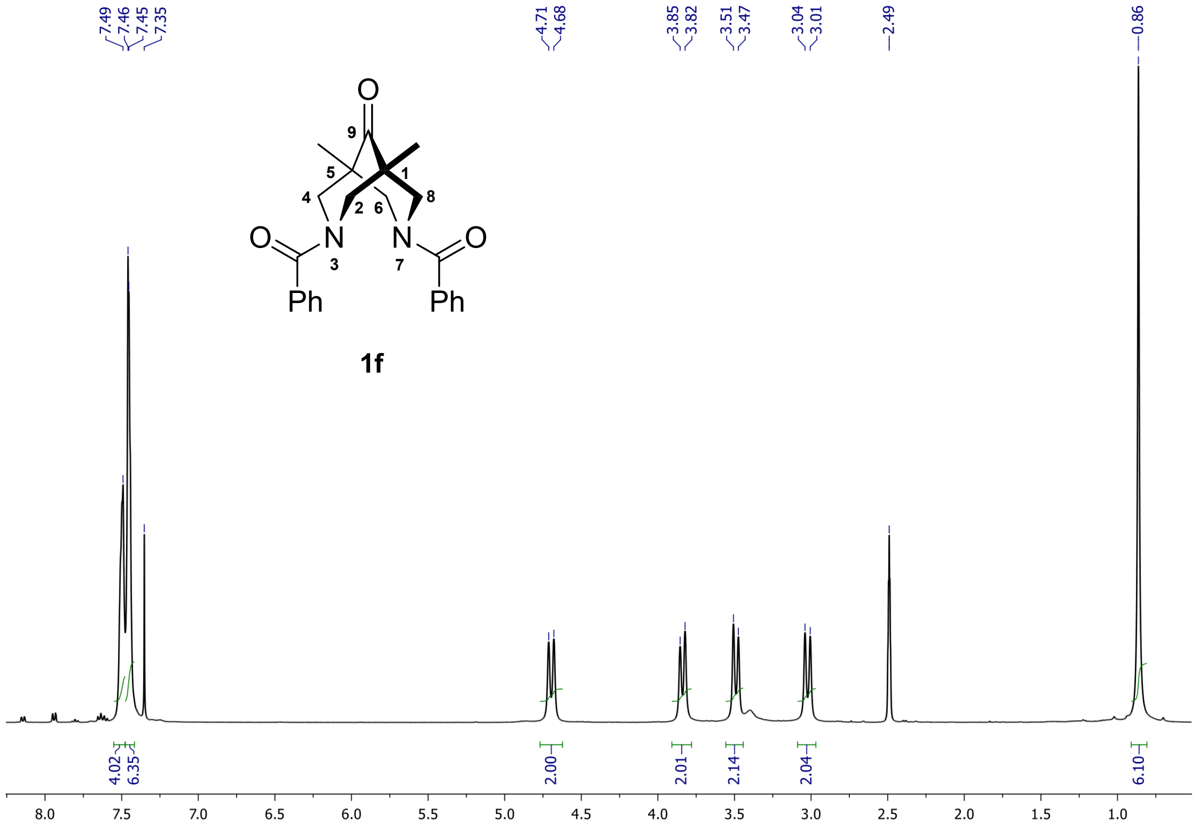

Figure S9. ^{1}H NMR spectrum of **1f** in DMSO-d_6.

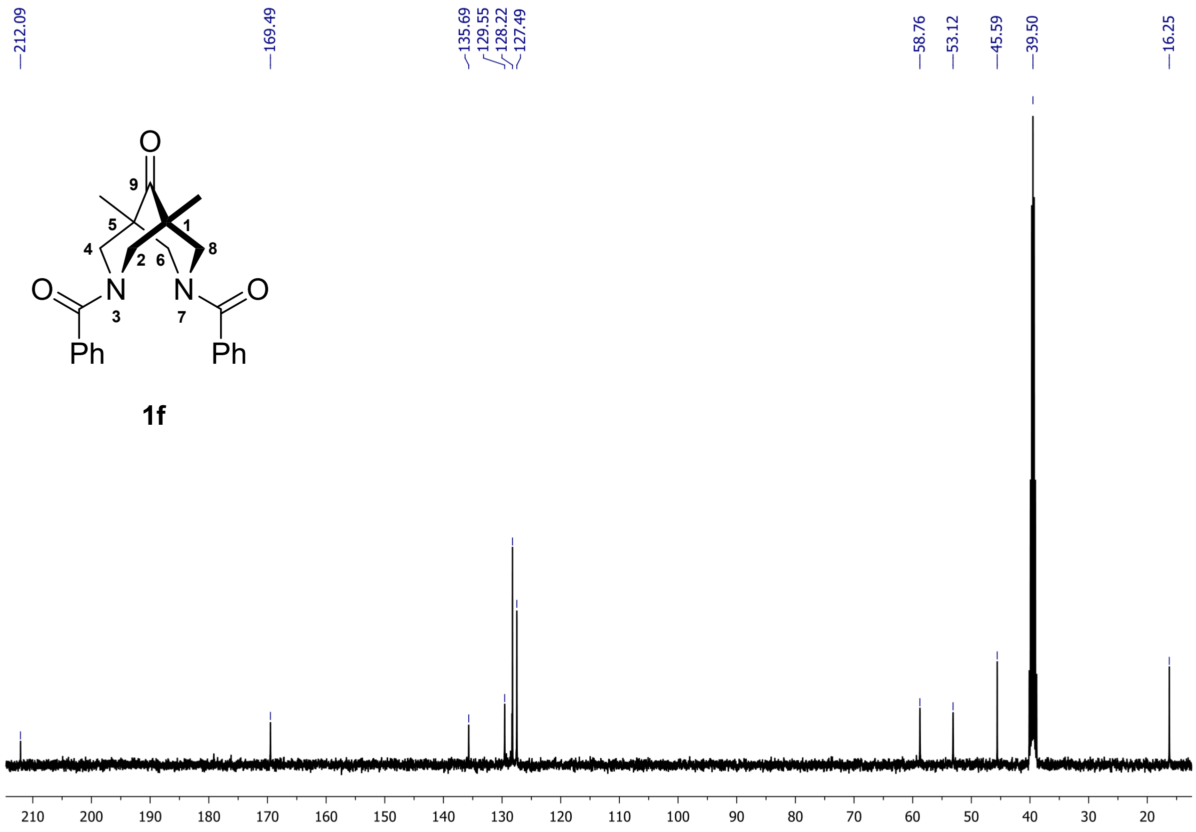

Figure S10. ^{13}C NMR spectrum of **1f** in DMSO-d$_6$.

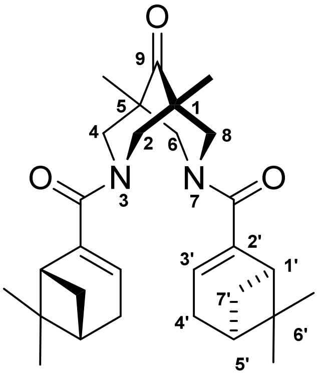

***(R,R)*-1g**

Compound **1g** (*anti*-). ^{1}H NMR (600 MHz, $CDCl_3$, δ/ppm, *J*/Hz): 0.93, 1.32 (both s, 6 H, $C^{6'}(CH_3)_2$); 1.02 (s, 6 H, $C^{1,5}CH_3$); 1.25 (br d, 2 H, $CH\mathit{H}^{7'}$); 2.12 (m, 2 H, $H^{5'}$); 2.42, 2.36 (*ABMX*-system, 4 H, $^2J_{HH}$ = 18.8, $^3J_{HH}$ = 3.0, 2.9, $H^{4'}$); 2.37 (br, 2 H, $H^{1'}$, derived from *HSQC*-spectrum); 2.49 (br, 2 H, $C\mathit{H}H^{7'}$); 2.8 (br, 2 H, $H^{4,8}$(ax));, 3.2 (br, 2 H, $H^{2,6}$(ax)); 4.2, 4.3 (both br, 1 H, $H^{2,6}$(eq)), 4.8 (br, 2 H, $H^{4,8}$(eq)); 5.92 (br s, 2 H, $H^{3'}$). ^{13}C NMR (150 MHz, $CDCl_3$, δ/ppm): 16.93 ($C^{1,5}\mathit{C}H_3$); 21.17 (br s), 25.96 ($C^{6'}(\mathit{C}H_3)_2$); 31.74, 31.75 ($CH_2^{4',7'}$); 37.9 (br, $C^{6'}$); 40.35 ($CH^{5'}$); 44.23 ($CH^{1'}$); 46.19 ($C^{1,5}$); 53.9 (br, $CH_2^{4,8}$); 58.5 (br, $CH_2^{2,6}$); 127.5, 128.3 (both br, $CH^{3'}$); 142.6 (br, $C^{2'}$); 169.9 (br, C(O)N); 212.91 (C^9=O).

Compound **1g** (*anti*-). ^{1}H NMR (500 MHz, $(CD_3)_2SO$, 25 °C, δ/ppm, *J*/Hz): 0.85 (br), 1.26 (s) (both 6 H, $C^{6'}(CH_3)_2$); 0.91 (s, 6 H, $C^{1,5}CH_3$); 1.15 (br, 2 H, $CH\mathit{H}^{7'}$); 2.07 (br m, 2 H, $H^{5'}$); 2.24 – 2.42 (set of br m, 8 H, $H^{1'}$, $H^{4'}$, $C\mathit{H}H^{7'}$); 2.9 (br, 2 H, $H^{4,8}$(ax));, 3.3 (br, overlapped with the signal of H_2O, $H^{2,6}$(ax)); 4.0, 4.2 (both br, 1 H, $H^{2,6}$(eq)), 4.6 (br, 2 H, $H^{4,8}$(eq)); 5.77 (br, 2 H, $H^{3'}$). ^{13}C NMR (125 MHz, $(CD_3)_2SO$, 25 °C, δ/ppm): 16.56 ($C^{1,5}\mathit{C}H_3$); 20.89 (br s), 25.79 ($C^{6'}(\mathit{C}H_3)_2$); 31.15 ($CH_2^{4',7'}$); 37.3 (br, $C^{6'}$); 39.69 ($CH^{5'}$); 43.53 ($CH^{1'}$); 45.46 ($C^{1,5}$); 53.0 (br, $CH_2^{4,8}$); 57.7 (br, $CH_2^{2,6}$); 126.0 (br, $CH^{3'}$); 142.0, 142.4 (both br, $C^{2'}$); 168.51 (C(O)N); 212.32 (C^{9}=O).

Compound **1g** (*anti*-). ^{1}H NMR (500 MHz, $(CD_3)_2SO$, 80 °C, δ/ppm, *J*/Hz): 0.88, 1.28 (both s, 6 H, $C^{6'}(CH_3)_2$); 0.93 (s, 6 H, $C^{1,5}CH_3$); 1.18 (d, 2 H, $^{2}J_{HH}$ = 8.8, $CH\mathit{H}^{7'}$); 2.10 (m, 2 H, $H^{5'}$); 2.29, 2.38 (*ABMX*-system, 4 H, $^{2}J_{HH}$ = 18.6, $^{3}J_{HH}$ = 3.0, 2.9, $H^{4'}$); 2.33 (br t, 2 H, $^{3}J_{HH}$ = 5.5, $CH^{1'}$); 2.42 (dt, 2 H, $^{2}J_{HH}$ = 8.8, $^{3}J_{HH}$ = 5.6, $C\mathit{H}H^{7'}$); 3.1 (br, overlapped with the signal of H_2O, $H^{2,4,6,8}$(ax)); 4.4 (br, 4 H, $H^{2,4,6,8}$ (eq)); 5.77 (m, 2 H, $H^{3'}$). ^{13}C NMR (125 MHz, $(CD_3)_2SO$, 80 °C, δ/ppm): 16.11 ($C^{1,5}\mathit{C}H_3$); 20.53, 25.48 ($C^{6'}(\mathit{C}H_3)_2$); 30.85 ($CH_2^{4',7'}$); 36.91 ($C^{6'}$); 39.67 ($CH^{5'}$); 43.53 ($CH^{1'}$); 45.15 ($C^{1,5}$); 55.3 (br, $CH_2^{2,4,6,8}$); 125.38 ($CH^{3'}$); 142.11 ($C^{2'}$); 168.25 (C(O)N); 211.56 (C^{9}=O).

Methyl group $C^{6'}(CH_3)$ at 0.93 ppm is directed toward double bond (see corresponding cross-peaks in *NOESY* spectrum).

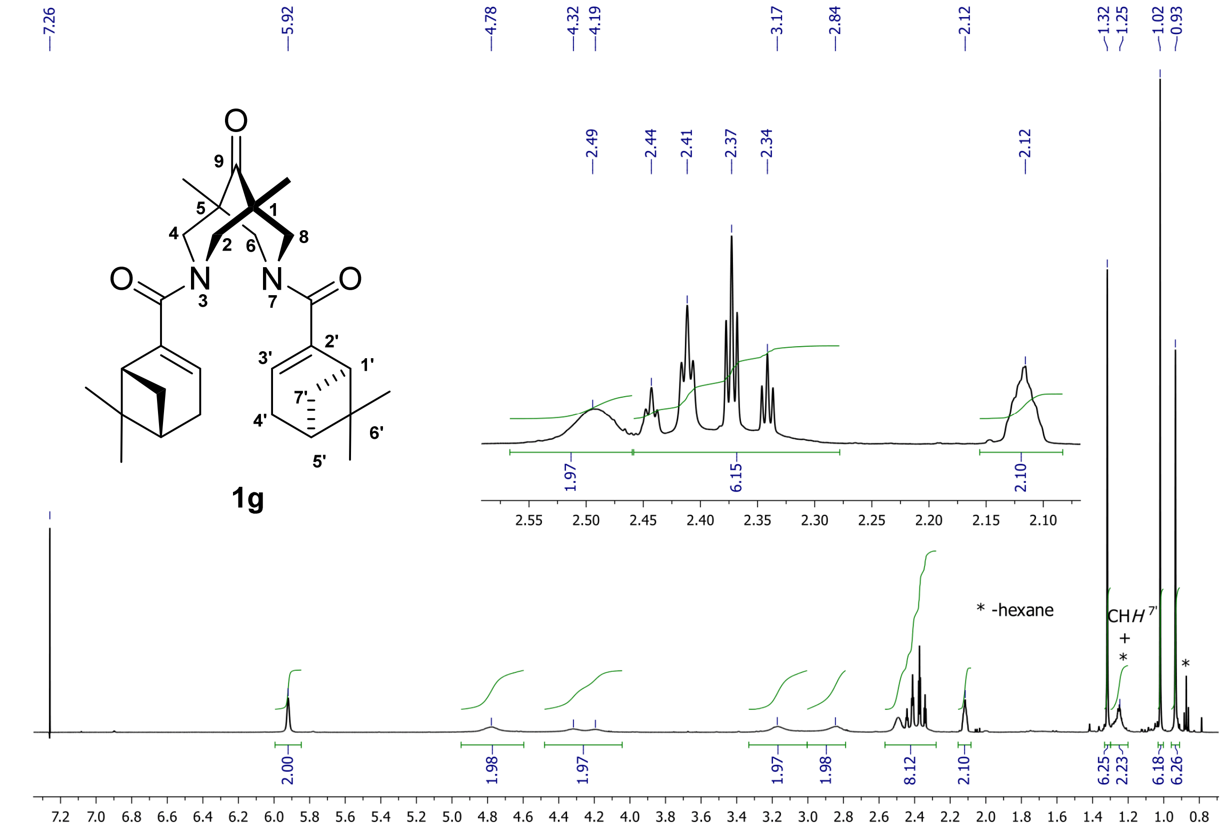

Figure S11. ^{1}H NMR spectrum of **1g** in $CDCl_3$.

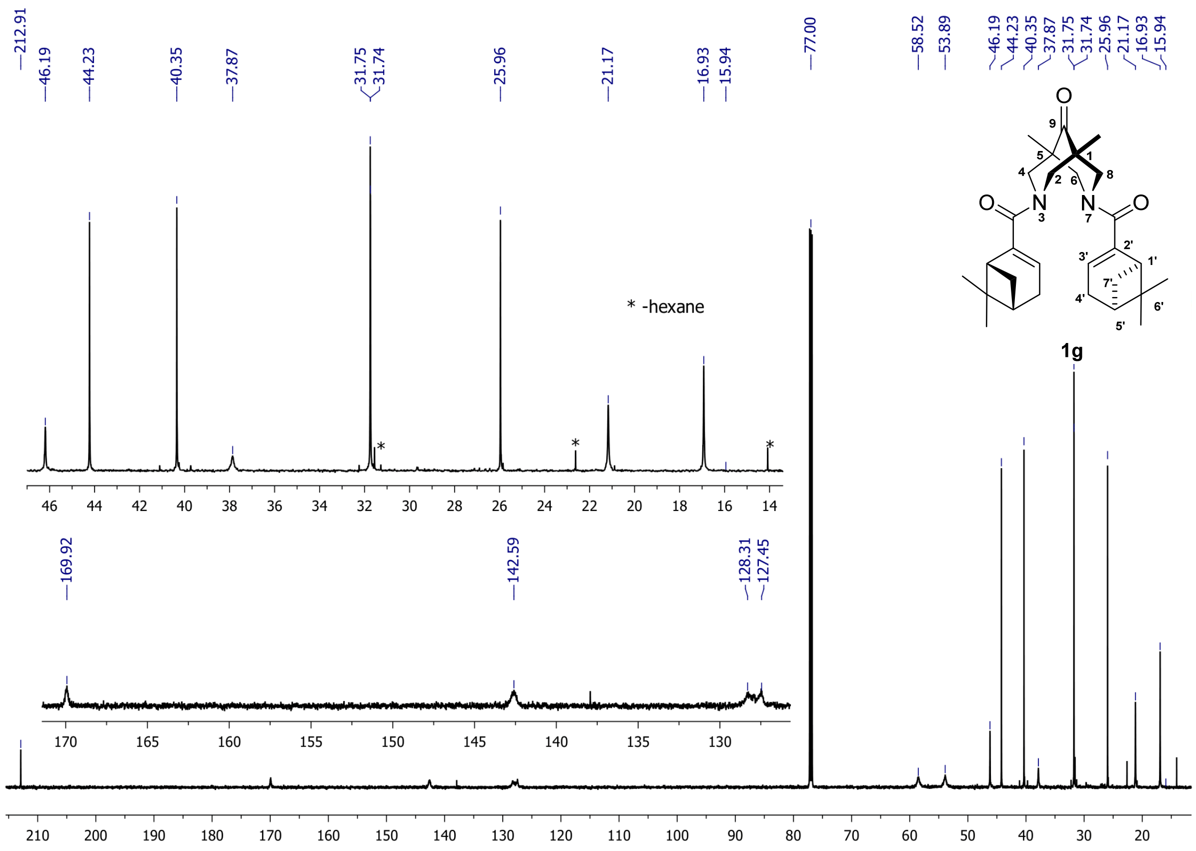

Figure S12. ^{13}C NMR spectrum of **1g** in $CDCl_3$.

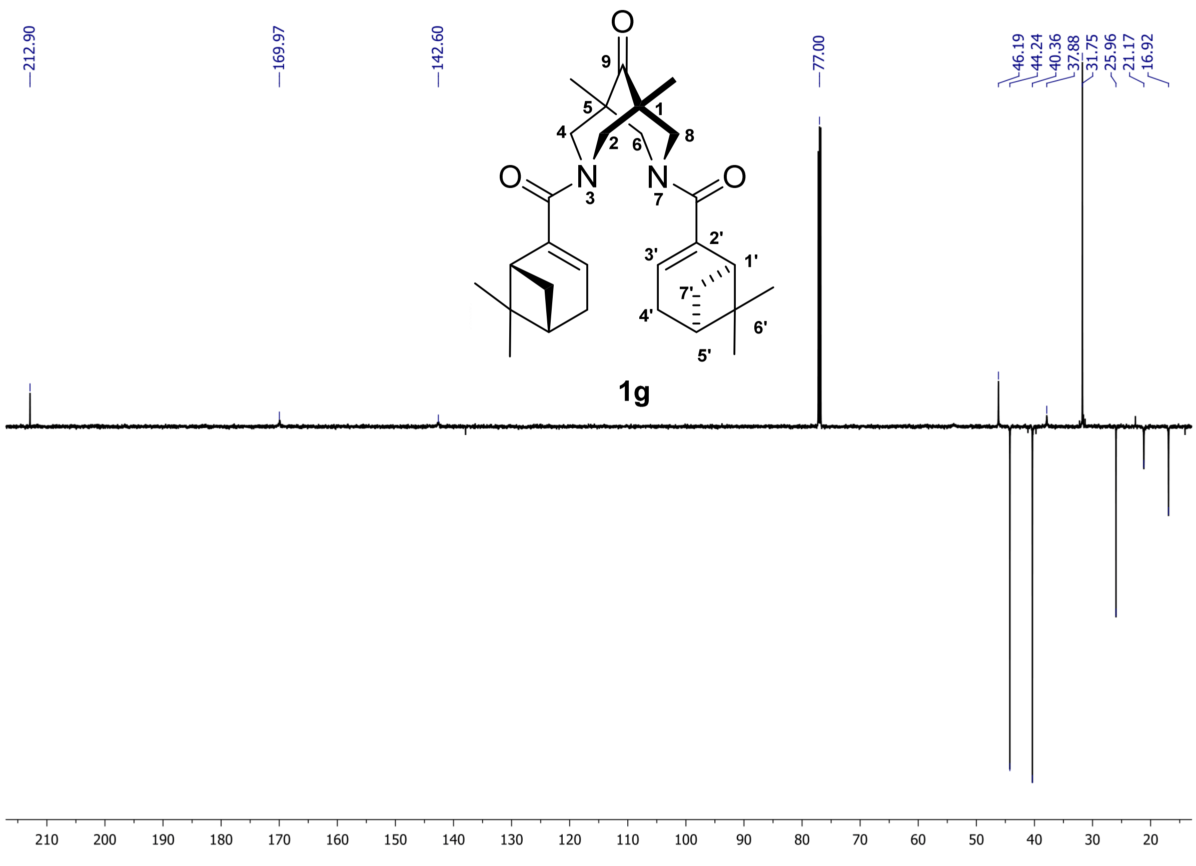

Figure S13. *APT* NMR spectrum of **1g** in $CDCl_3$.

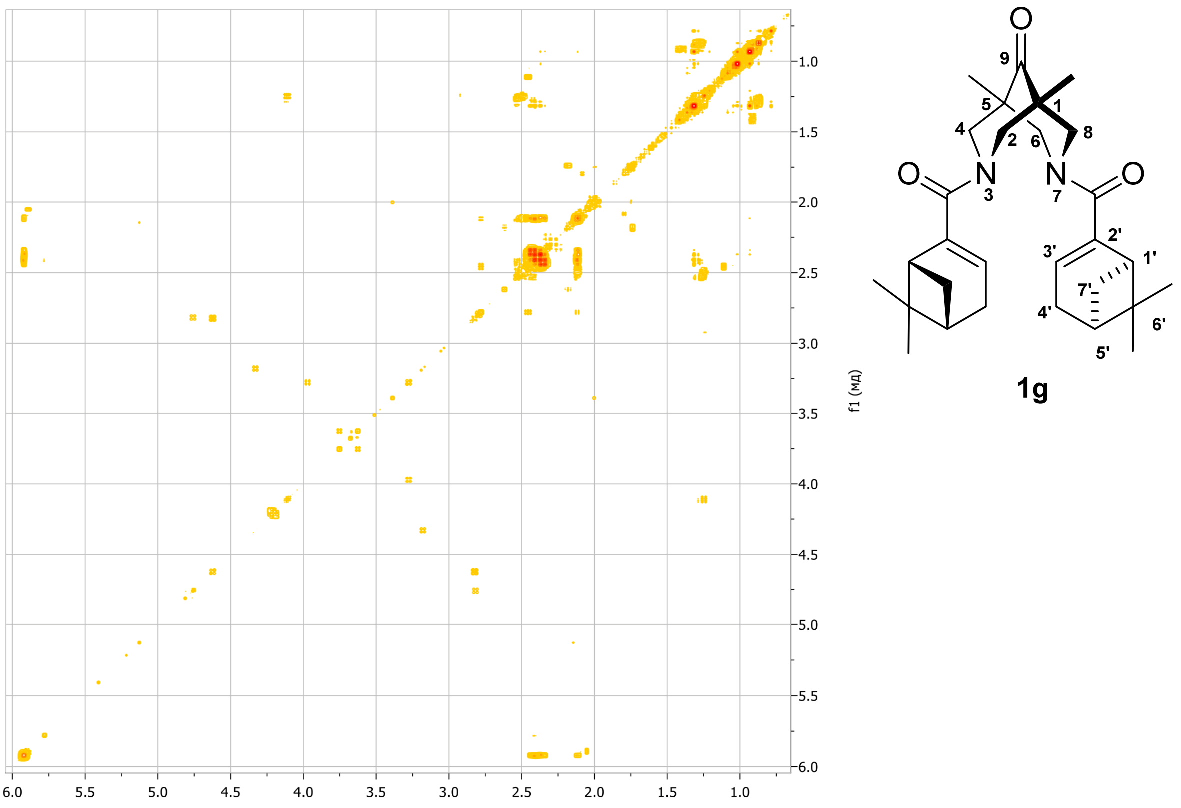

Figure S14. *COSY* NMR spectrum of **1g** in $CDCl_3$

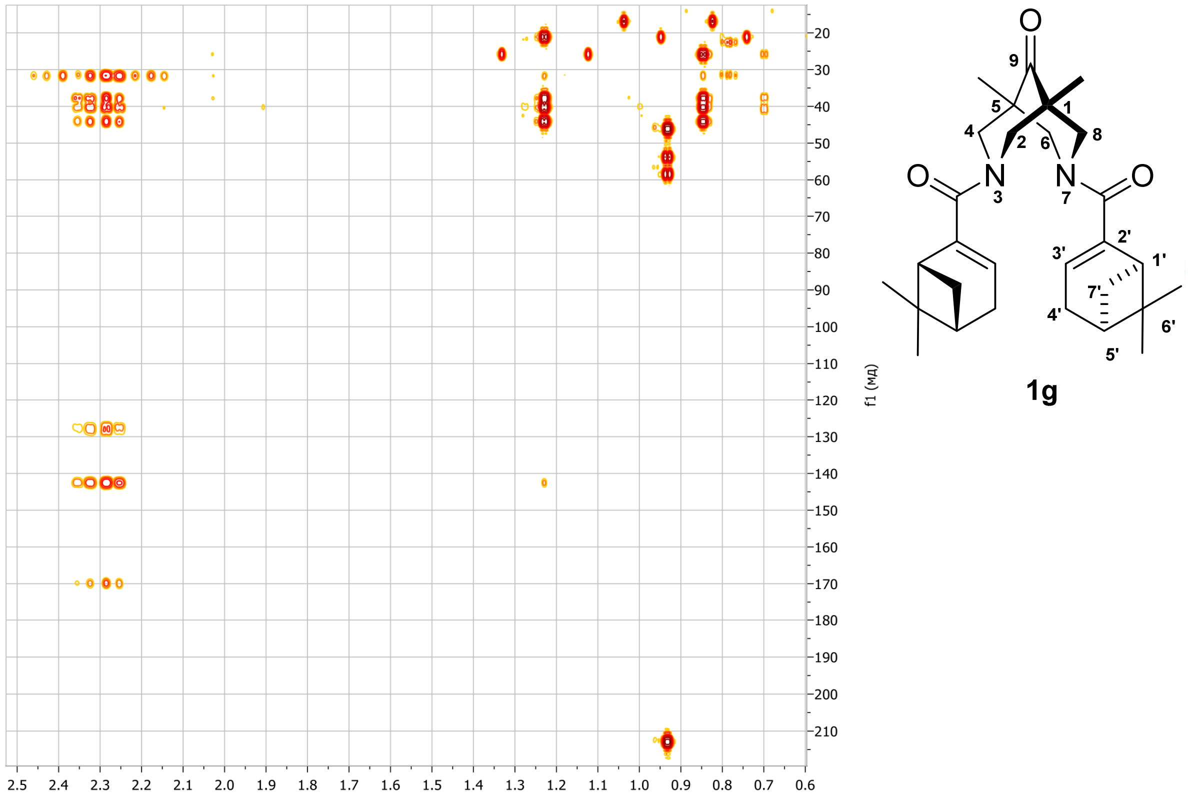

Figure S15. *HSQC* NMR spectrum of **1g** in CDCl3

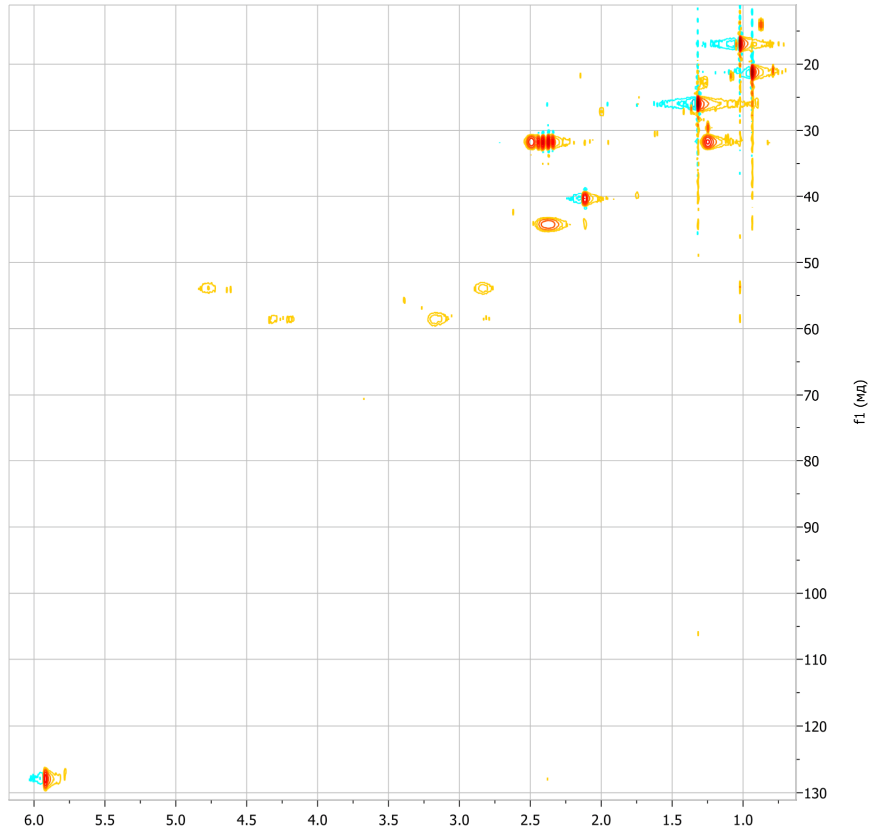

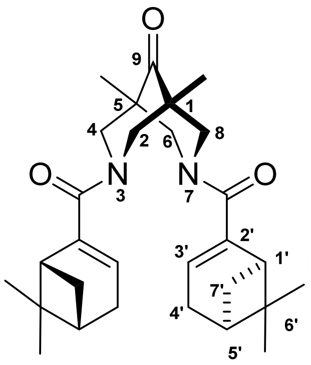

1g

Figure S16. *HMBC* NMR spectrum of **1g** in CDCl3.

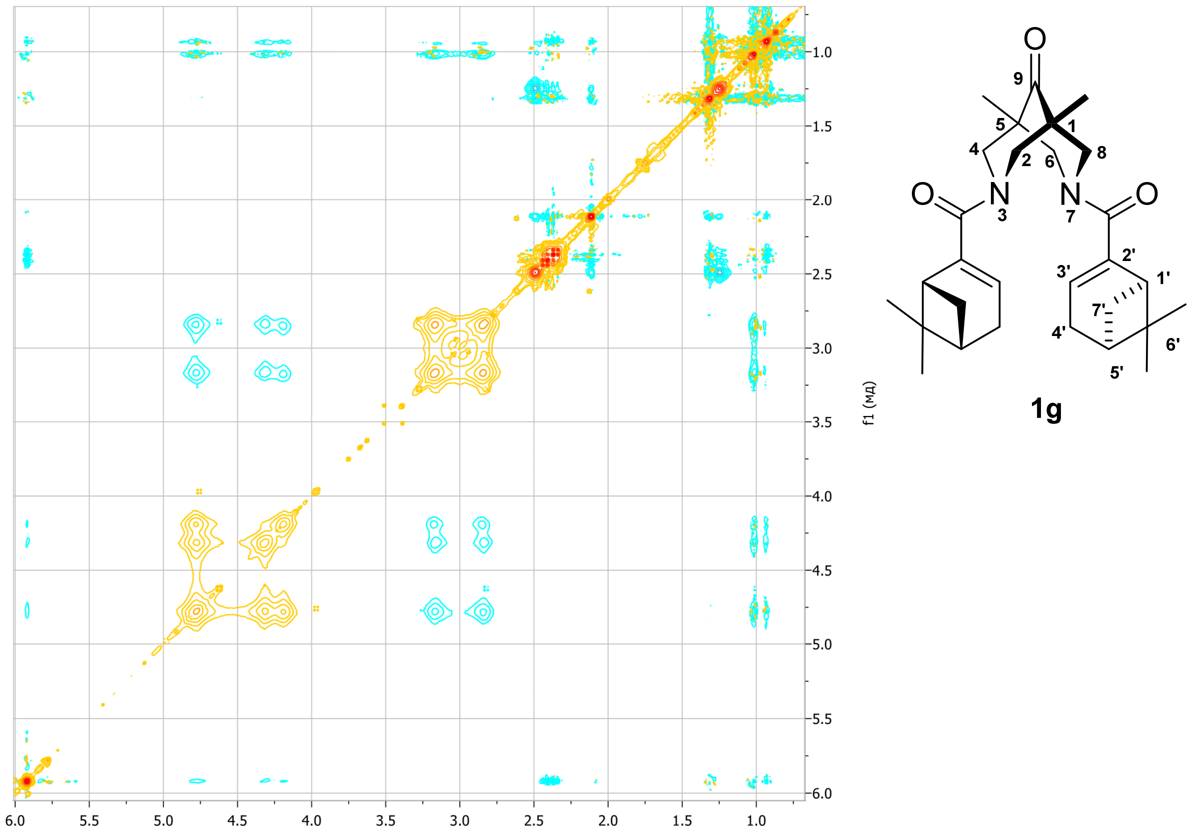

Figure S17. *NOESY* NMR spectrum of **1g** in $CDCl_3$.

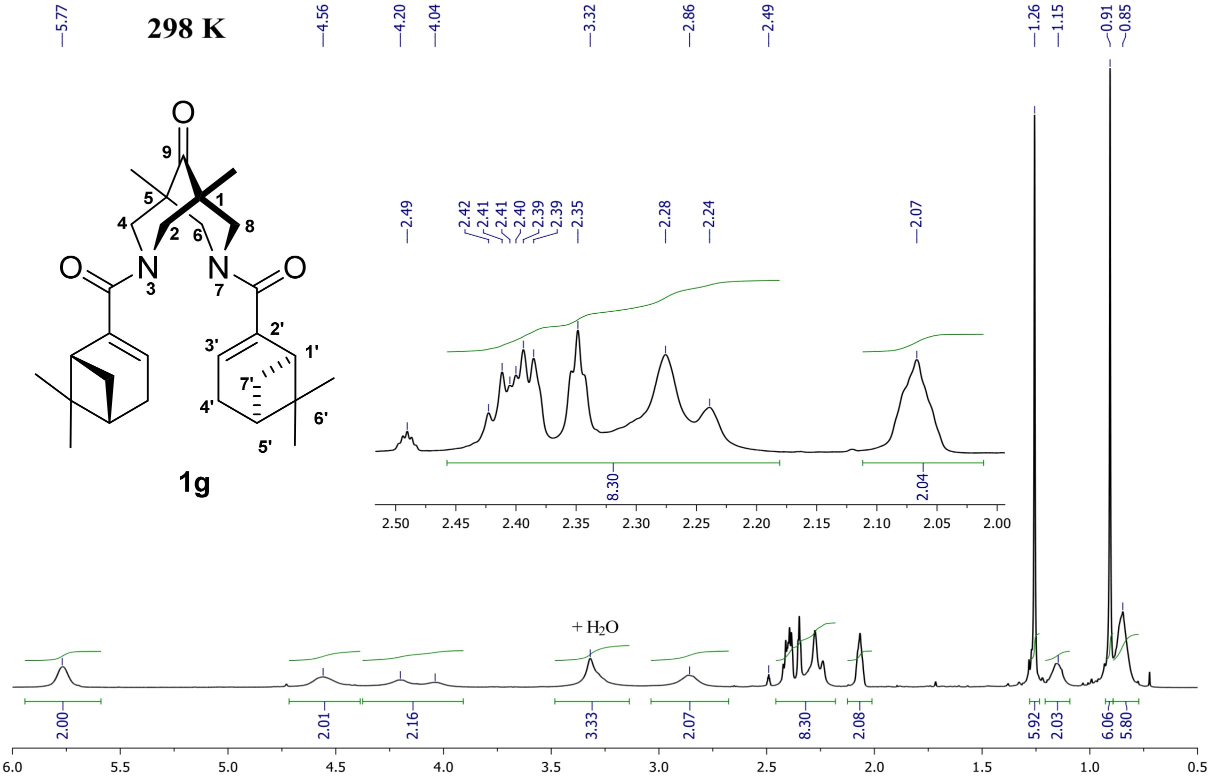

Figure S18. ^{1}H NMR spectrum of **1g** in DMSO-d_6 at 298 K.

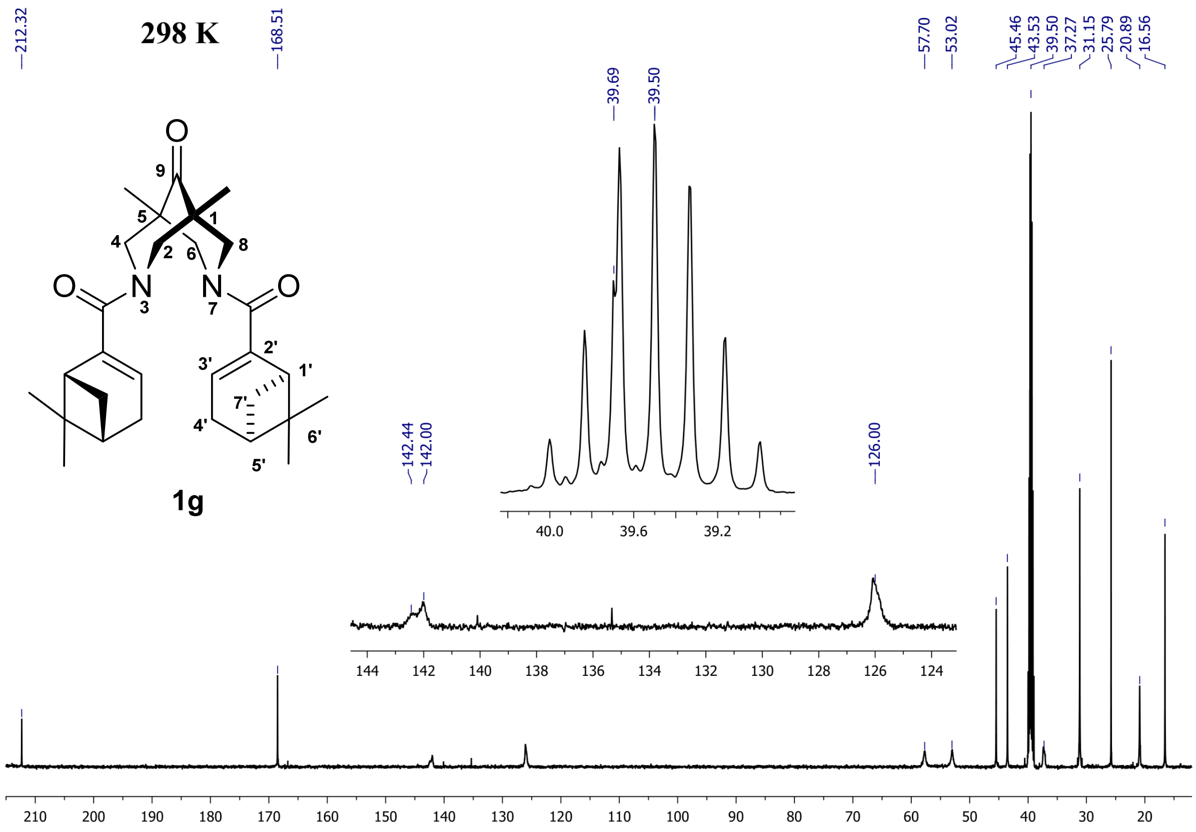

Figure S19. ^{13}C NMR spectrum of **1g** in DMSO-d6 at 298 K.

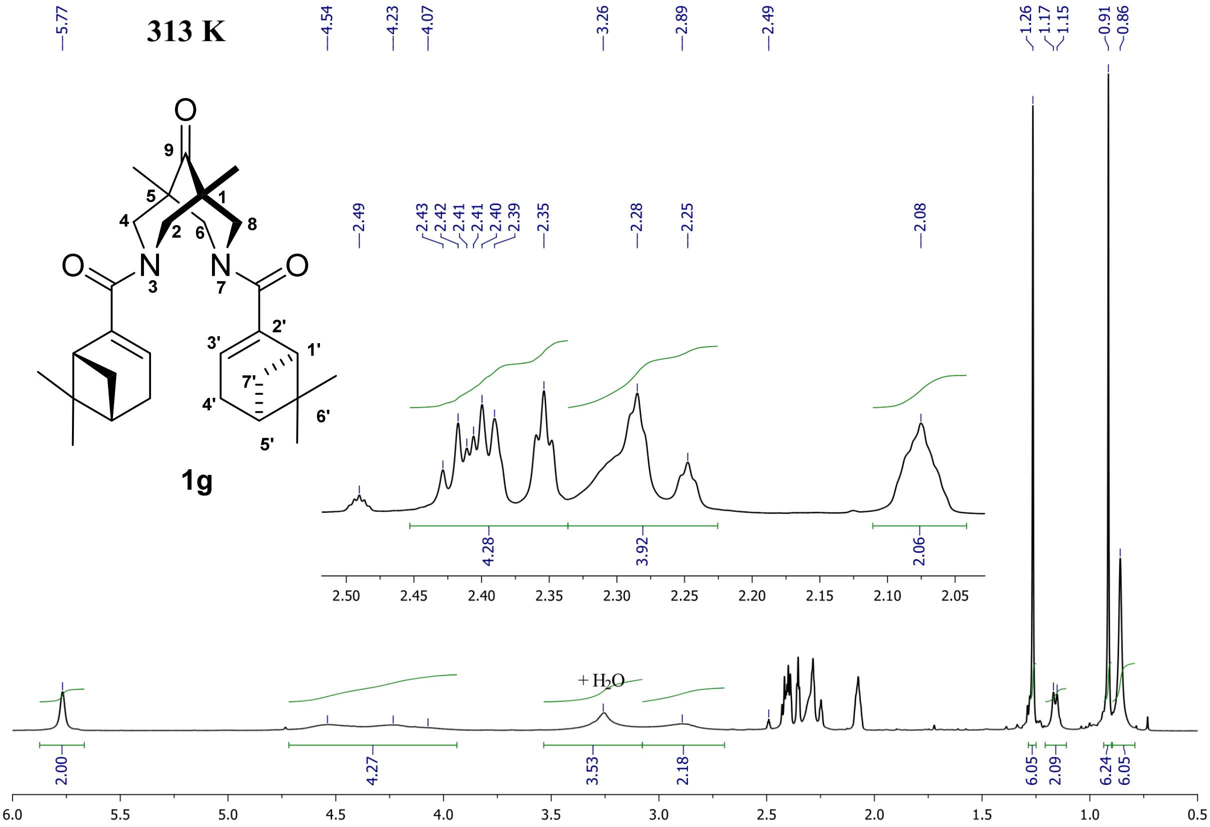

Figure S20. ^{1}H NMR spectrum of **1g** in DMSO-d6 at 313 K.

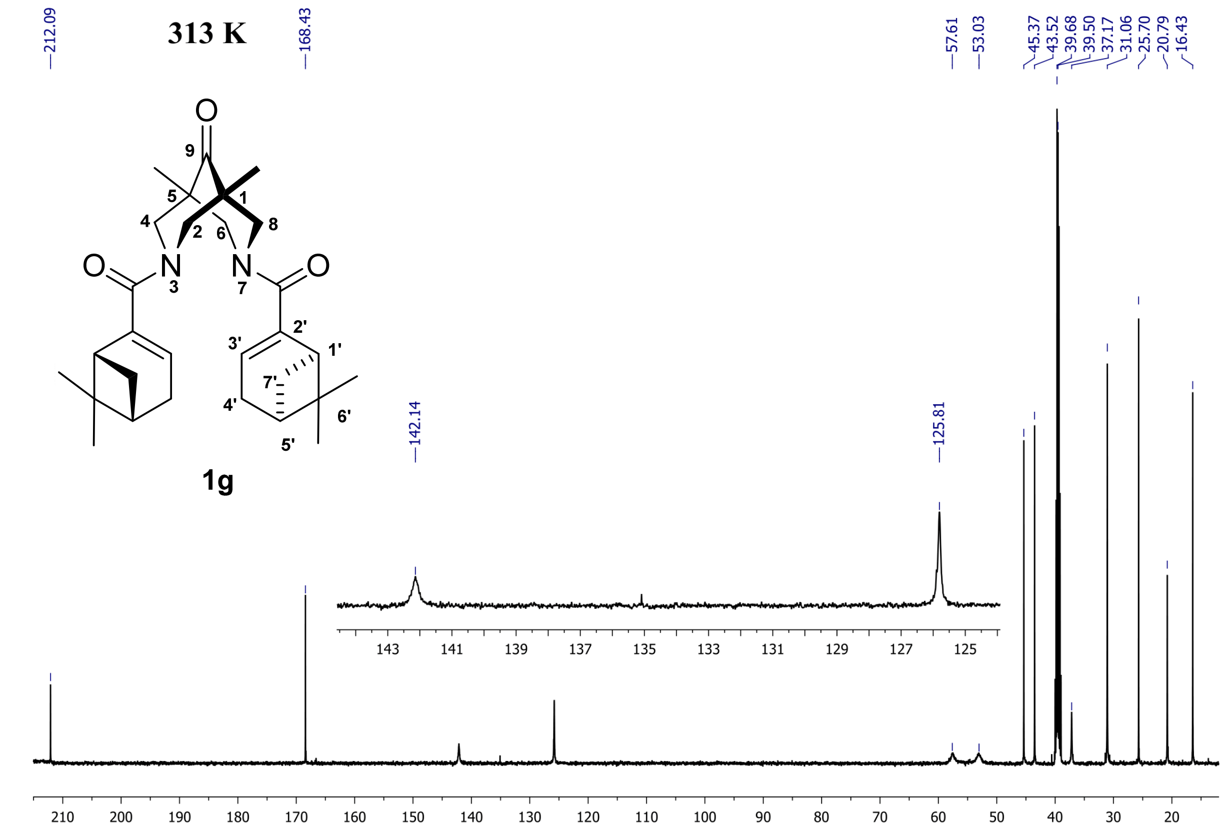

Figure S21. ^{13}C NMR spectrum of **1g** in DMSO-d6 at 313 K.

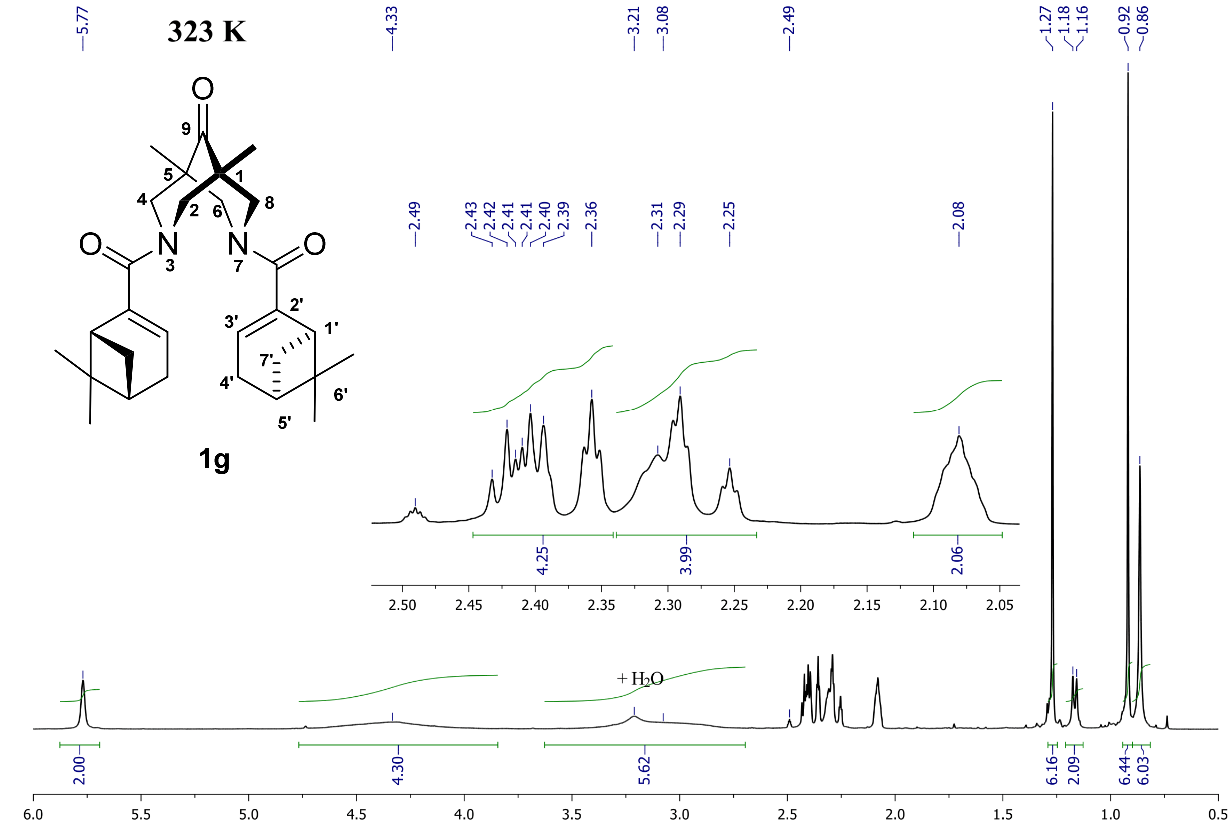

Figure S22. ^{1}H NMR spectrum of **1g** in DMSO-d6 at 323 K.

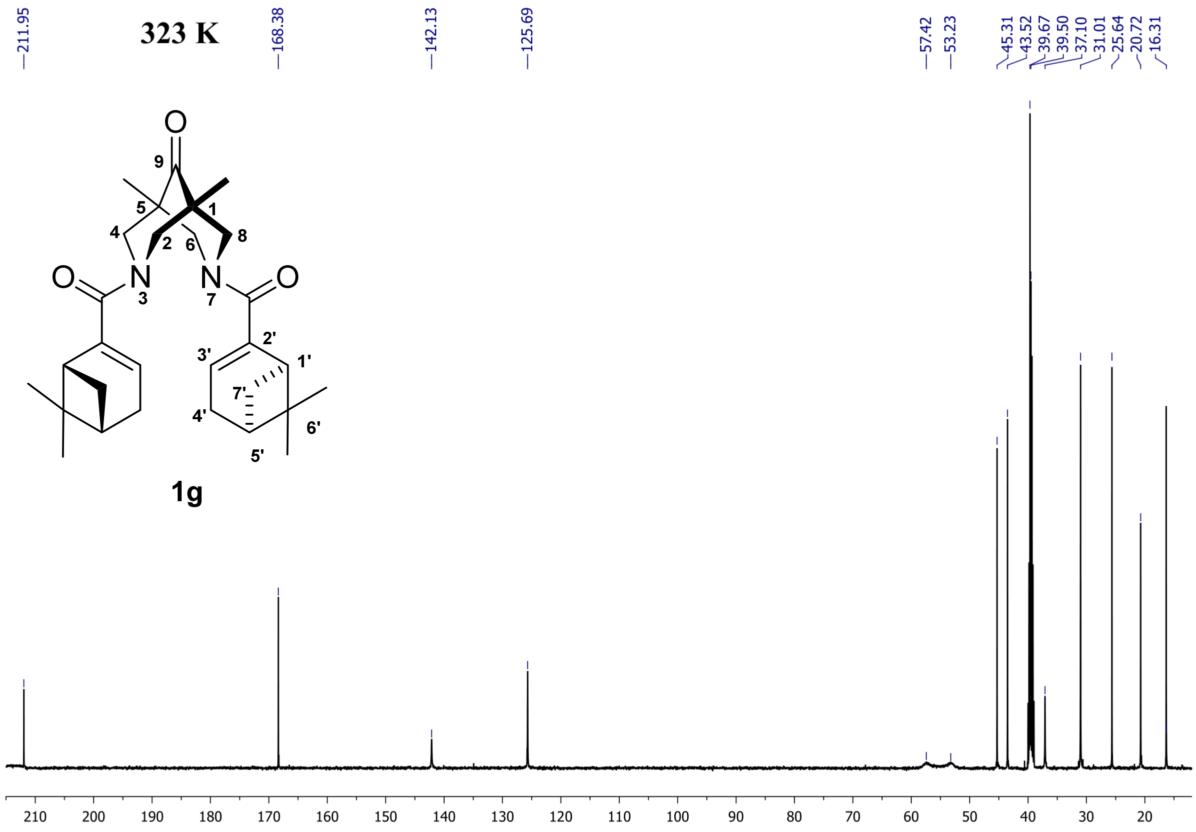

Figure S23. ^{13}C NMR spectrum of **1g** in DMSO-d$_6$ at 323 K.

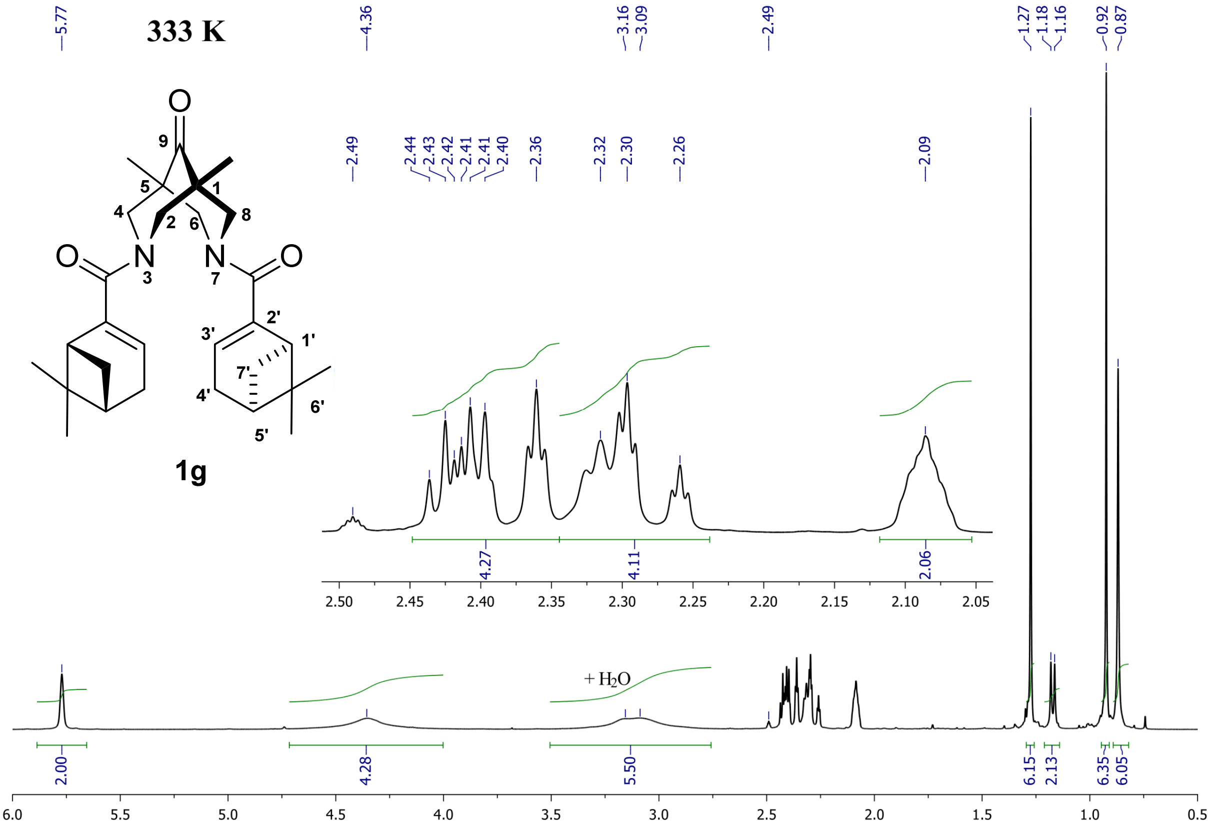

Figure S24. ^{1}H NMR spectrum of **1g** in DMSO-d$_6$ at 333 K.

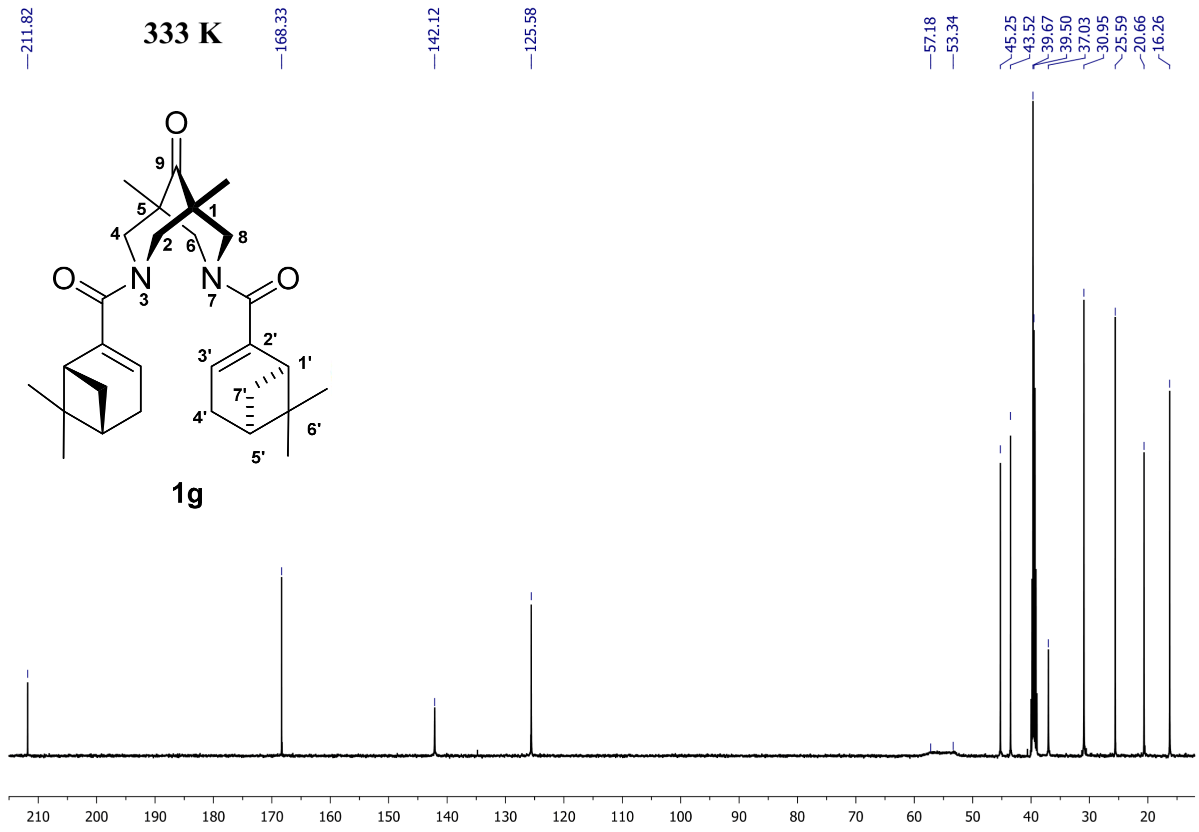

Figure S25. ^{13}C NMR spectrum of **1g** in DMSO-d6 at 333 K.

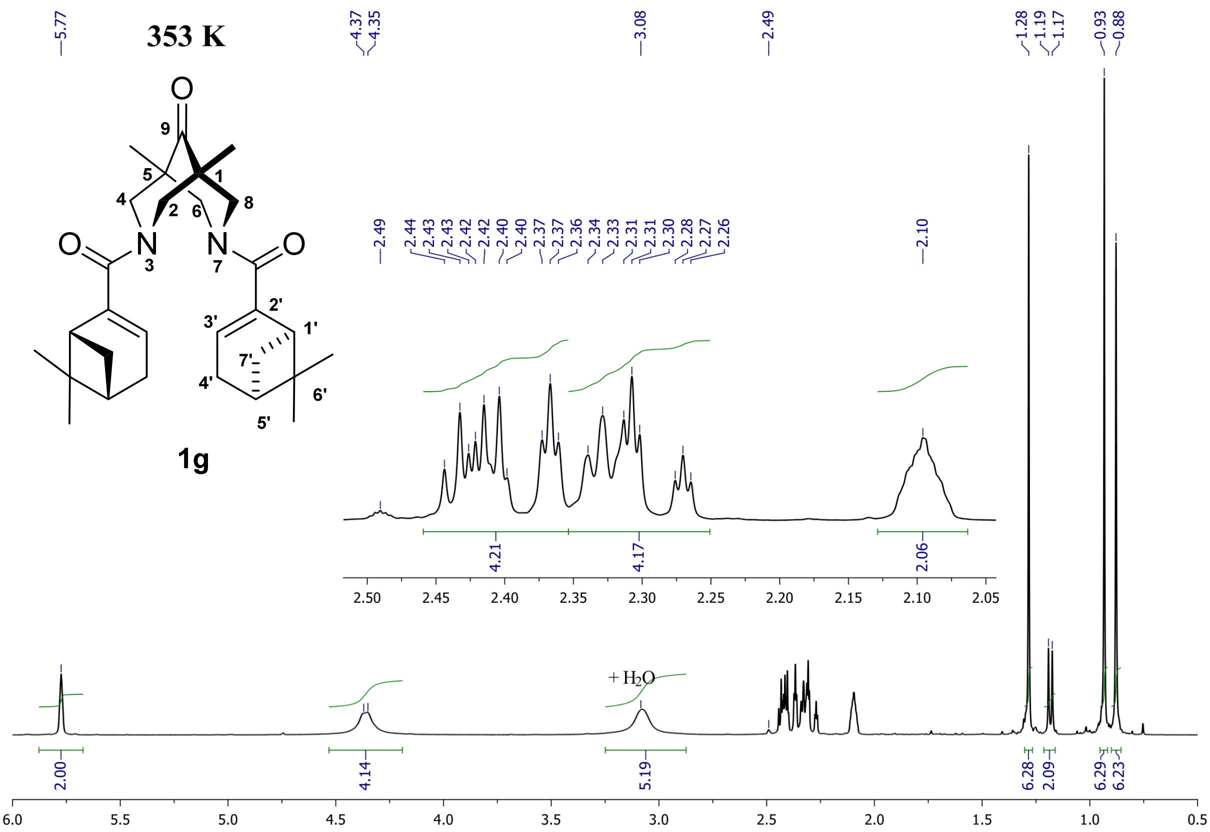

Figure S26. ^{1}H NMR spectrum of **1g** in DMSO-d6 at 353 K.

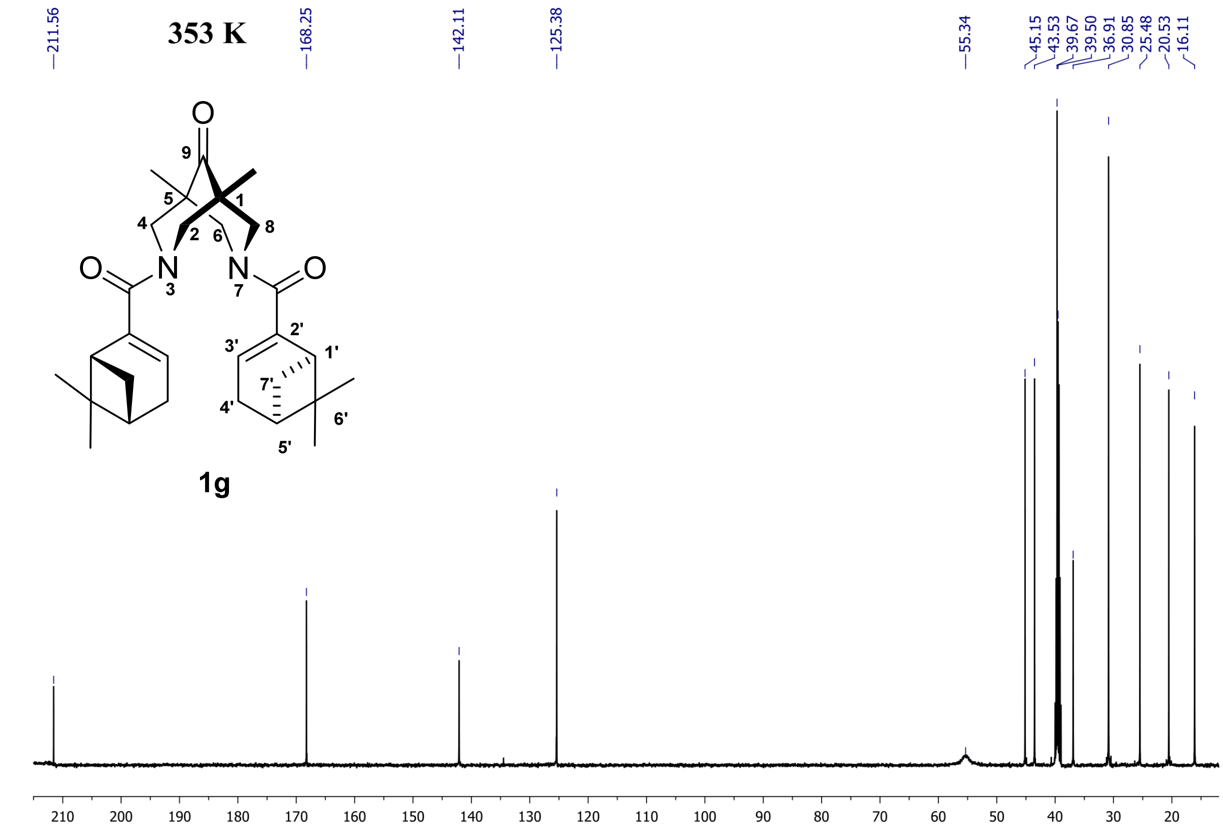

Figure S27. ^{13}C NMR spectrum of **1g** in DMSO-d$_6$ at 353 K.

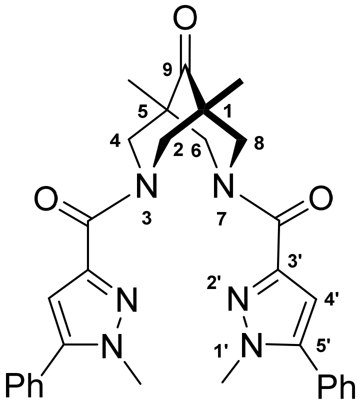

1h

Compound **1h** (*syn-*/*anti-* = 1.18). ^{1}H NMR (400 MHz, $(CD_3)_2SO$, δ/ppm, *J*/Hz): 0.95, 1.05 (both s, CCH_3 (*syn-*)); 0.96 (s, CCH_3 (*anti-*)); 3.00 (d, $^{2}J_{HH}$ = 13.0, H(ax) (*anti-*)); 3.01 (d, $^{2}J_{HH}$ = 12.9, H(ax) (*syn-*)); 3.42 (d, $^{2}J_{HH}$ = 13.6, H(ax) (*syn-*)); 3.47 (d, $^{2}J_{HH}$ = 13.9, H(ax) (*anti-*)); 3.66 (s, NCH_3 (*syn-*)); 3.89 (s, NCH_3 (*anti-*)); 4.80 (d, $^{2}J_{HH}$ = 13.2, H(eq) (*anti-*)); 4.87 (d, $^{2}J_{HH}$ = 13.9, H(eq) (*anti-*)); 4.91 (d, $^{2}J_{HH}$ = 12.9, H(eq) (*syn-*)); 5.38 (d, $^{2}J_{HH}$ = 13.3, H(eq) (*syn-*)); 6.39 (s, $H^{4'}$ (*syn-*)); 6.75 (s, $H^{4'}$ (*anti-*)); 7.28 – 7.61 (set of m) (Ph (*syn-*, *anti-*)).

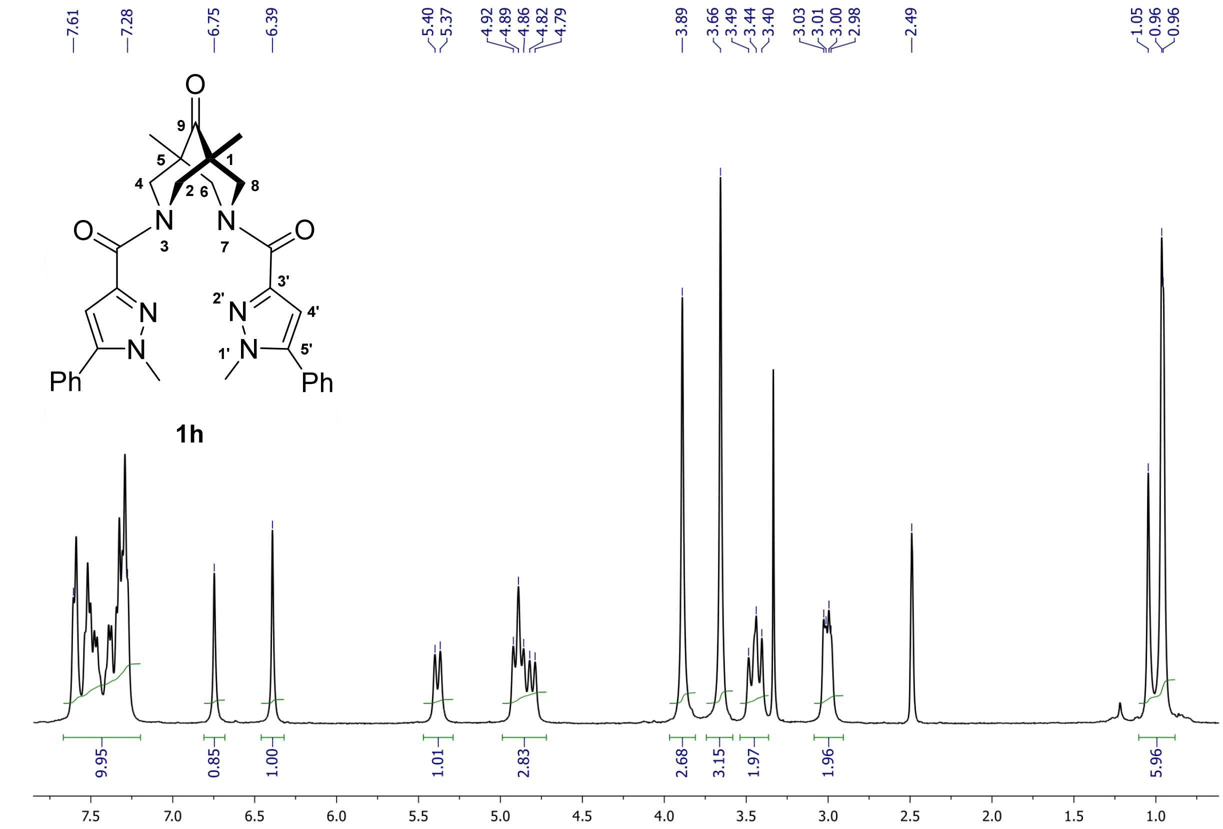

Figure S28. ^{1}H NMR spectrum of **1h** in DMSO-d$_6$.

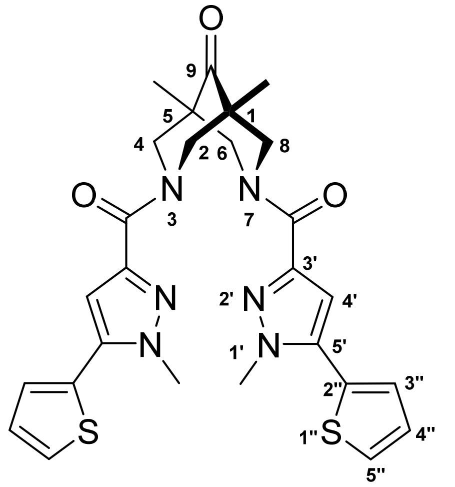

1i

Compound **1i** (*syn*-/*anti*- = 1.00). ^{1}H NMR (500 MHz, $(CD_3)_2SO$, 25 °C, δ/ppm, *J*/Hz): 0.94, 1.04 (both s, CCH_3 (*syn*-)); 0.95 (s, CCH_3 (*anti*-)); 2.98 (d, $^{2}J_{HH}$ = 13.5), 3.00 (d, $^{2}J_{HH}$ = 13.2); 3.40 (d, $^{2}J_{HH}$ = 13.3), 3.46 (d, $^{2}J_{HH}$ = 13.5) (H(ax) (*syn*-, *anti*-)); 3.76 (s, NCH_3 (*syn*-)); 3.97 (s, NCH_3 (*anti*-)); 4.77 (d, $^{2}J_{HH}$ = 13.5), 4.85 (d, $^{2}J_{HH}$ = 13.7), 4.88 (d, $^{2}J_{HH}$ = 13.2) (H(eq) (*syn*-, *anti*-)); 5.33 (d, $^{2}J_{HH}$ = 13.3, H(eq) (*syn*-)); 6.44 (s, $H^{4'}$ (*syn*-)); 6.77 (s, $H^{4'}$ (*anti*-)); 7.09 (br t, $^{3}J_{HH}$ = 4.2), 7.23 (br t, $^{3}J_{HH}$ = 4.3) ($H^{4''}$ (*syn*-, *anti*-)); 7.21 (br d, $^{3}J_{HH}$ = 3.1, $H^{3''}$ (*syn*-)); 7.49 (br d, $^{3}J_{HH}$ = 2.9, $H^{3''}$ (*anti*-)); 7.62 (br d, $^{3}J_{HH}$ = 4.9, $H^{5''}$(*syn*-)); 7.75 (br d, $^{3}J_{HH}$ = 4.9, $H^{5''}$ (*anti*-)). ^{13}C NMR (125 MHz, $(CD_3)_2SO$, 25 °C, δ/ppm): 16.38 (CCH_3 (*anti*-)); 16.46, 16.68

(CCH$_3$ (*syn*-)); 38.27, 38.35 (NCH$_3$ (*syn*-, *anti*-)); 45.63, 45.87 ($C^{1,5}$ (*syn*-)); 46.05 ($C^{1,5}$ (*anti*-)); 53.20, 57.69 (CH$_2$N (*anti*-)); 53.99, 56.76 (CH$_2$N (*syn*-)); 108.72, 108.88 ($CH^{4'}$ (*syn*-, *anti*-)); 127.35, 127.58,127.80, 128.11, 128.31 ($CH^{3'',4'',5''}$ (*syn*-, *anti*-)); 129.57, 129.83 ($C^{2''}$ (*syn*-, *anti*-)); 136.46, 136.61 ($C^{5'}$ (*syn*-, *anti*-)); 145.32 ($C^{3'}$ (*syn*-, *anti*-)); 160.98, 161.91 (C(O)N (*syn*- , *anti*-)); 212.11, 212.33 (C^9=O (*syn*-, *anti*-)).

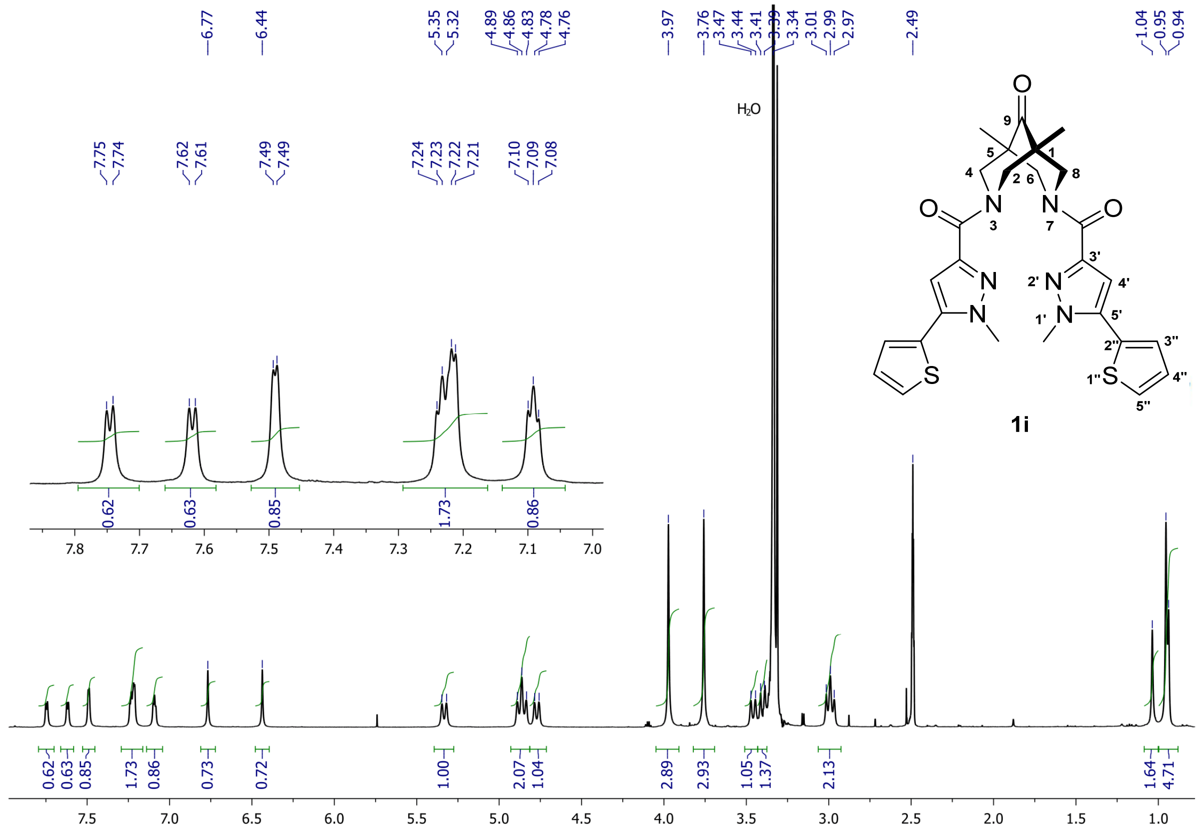

Figure S29. ^{1}H NMR spectrum of **1i** in DMSO-d$_6$.

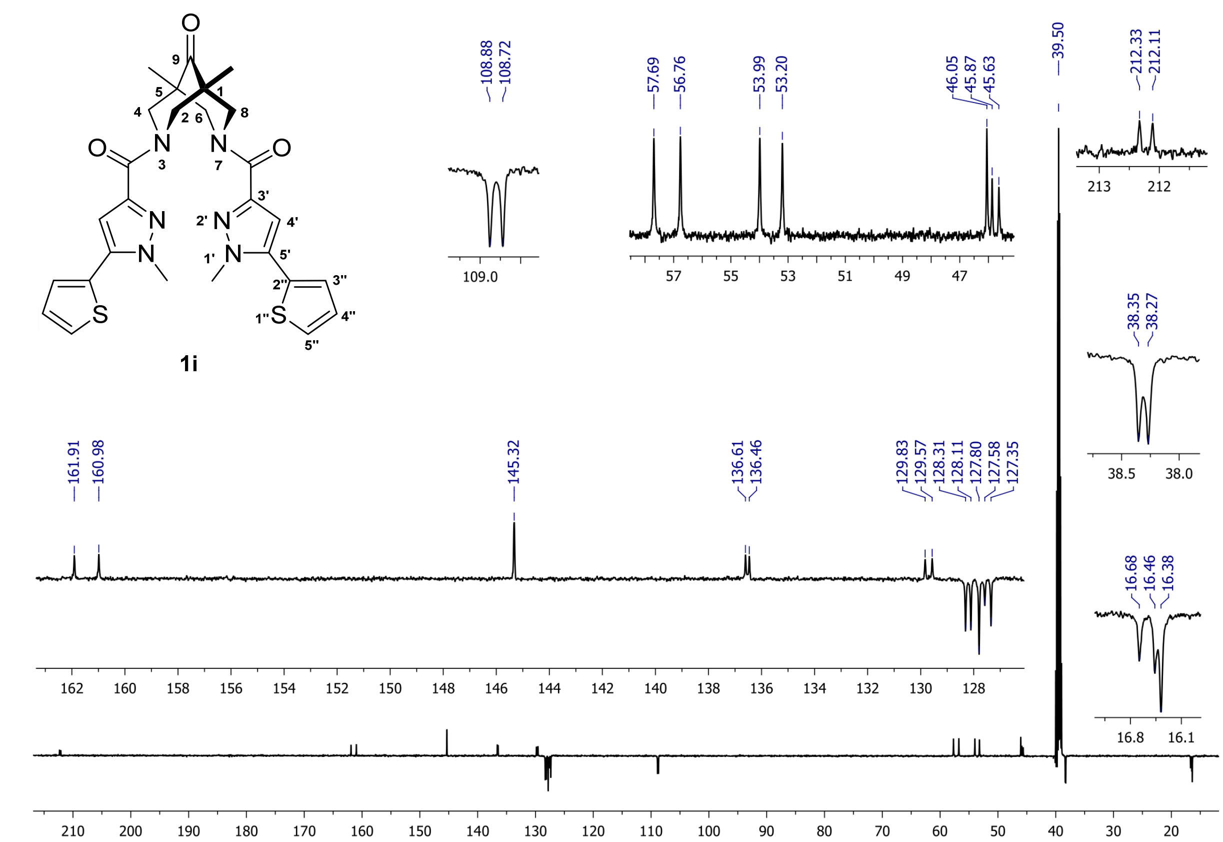

Figure S30. *APT* NMR spectrum of **1i** in DMSO-d$_6$.

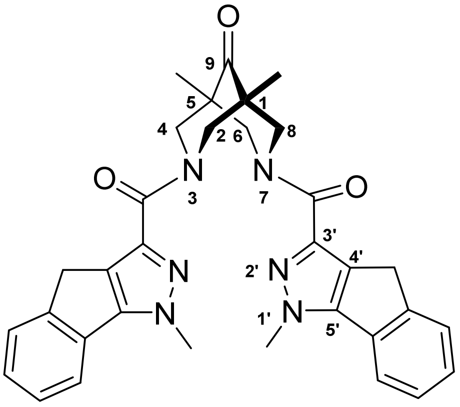

1j

Compound **1j** (*syn-/anti-* = 2.33). ^{1}H NMR (500 MHz, $(CD_3)_2SO$, 25 °C, δ/ppm, *J*/Hz): 0.97 (s, CCH_3 (*syn-*, *anti-*)); 1.04 (s, CCH_3 (*syn-*)); 2.98 (d, $^2J_{HH}$ = 13.6, H(ax) (*anti-*)); 2.99 (d, $^2J_{HH}$ = 13.4, H(ax) (*syn-*)), 3.05, 3.27 (*AB*-system, $^2J_{HH}$ = 21.6, CH_2 (*syn-*)); the doublet of second H(ax) (*syn-*)) is overlapped with the signal of H_2O; 3.46 (d, $^2J_{HH}$ = 13.6, H(ax) (*anti-*)); 3.63, 3.75 (*AB*-system, $^2J_{HH}$ = 21.7, CH_2 (*anti-*)); 3.82 (s, NCH_3 (*syn-*)); 4.15 (s, NCH_3 (*anti-*)); 4.75 (d, $^2J_{HH}$ = 13.6, H(eq) (*anti-*)); 4.91 (d, $^2J_{HH}$ = 13.2, H(eq) (*syn-*)); 5.11 (d, $^2J_{HH}$ = 13.5, H(eq) (*anti-*)); 5.83 (d, $^2J_{HH}$ = 13.4, H(eq) (*syn-*)); 6.92 (t, $^3J_{HH}$ = 7.5, H(Ar) (*syn-*)); 7.05 (d, $^3J_{HH}$ = 7.5, H(Ar) (*syn-*)); 7.10 (t, $^3J_{HH}$ = 7.5, H(Ar) (*syn-*)); 7.28 (d, $^3J_{HH}$ = 7.6, H(Ar) (*syn-*)); 7.31 (t, $^3J_{HH}$ = 7.5, H(Ar) (*anti-*)); 7.41 (t, $^3J_{HH}$ = 7.5, H(Ar) (*anti-*)); 7.56 (d, $^3J_{HH}$ = 7.5, H(Ar)

(*anti*-)); 7.78 (d, $^3J_{HH}$ = 7.6, H(Ar) (*anti*-)). ^{13}C NMR (125 MHz, $(CD_3)_2SO$, 25 °C, δ/ppm): 16.52 (CCH_3 (*syn*-, *anti*-)); 16.79 (CCH_3 (*syn*-)); 29.26 (CH_2 (*syn*-)); 29.72 (CH_2 (*anti*-)); 37.74 (NCH_3 (*syn*-)); 38.10 (NCH_3 (*anti*-)); 45.68, 46.19 ($C^{1,5}$ (*syn*-)); 46.31 ($C^{1,5}$ (*anti*-)); 53.22, 57.94 (CH_2N (*anti*-)); 53.91, 56.17 (CH_2N (*syn*-)); 118.15, 125.56, 125.71, 126.23 (CH(Ar) (*syn*-)); 118.94, 126.32, 126.39, 126.80 (CH(Ar) (*anti*-)); 128.92, 131.14 (C(Ar) (*anti*-)); 129.85, 130.35 (C(Ar) (*syn*-)); 139.32 ($C^{3'}$ (*syn*-)); 139.58 ($C^{3'}$ (*anti*-)); 147.89, 148.22 ($C^{4',5'}$ (*syn*-)); 148.64, 148.81 ($C^{4',5'}$ (*anti*-)); 160.78 (C(O)N (*syn*-)); 161.66 (C(O)N (*anti*-)); 212.43 (C^9=O (*anti*-)); 212.55 (C^9=O (*syn*-)).

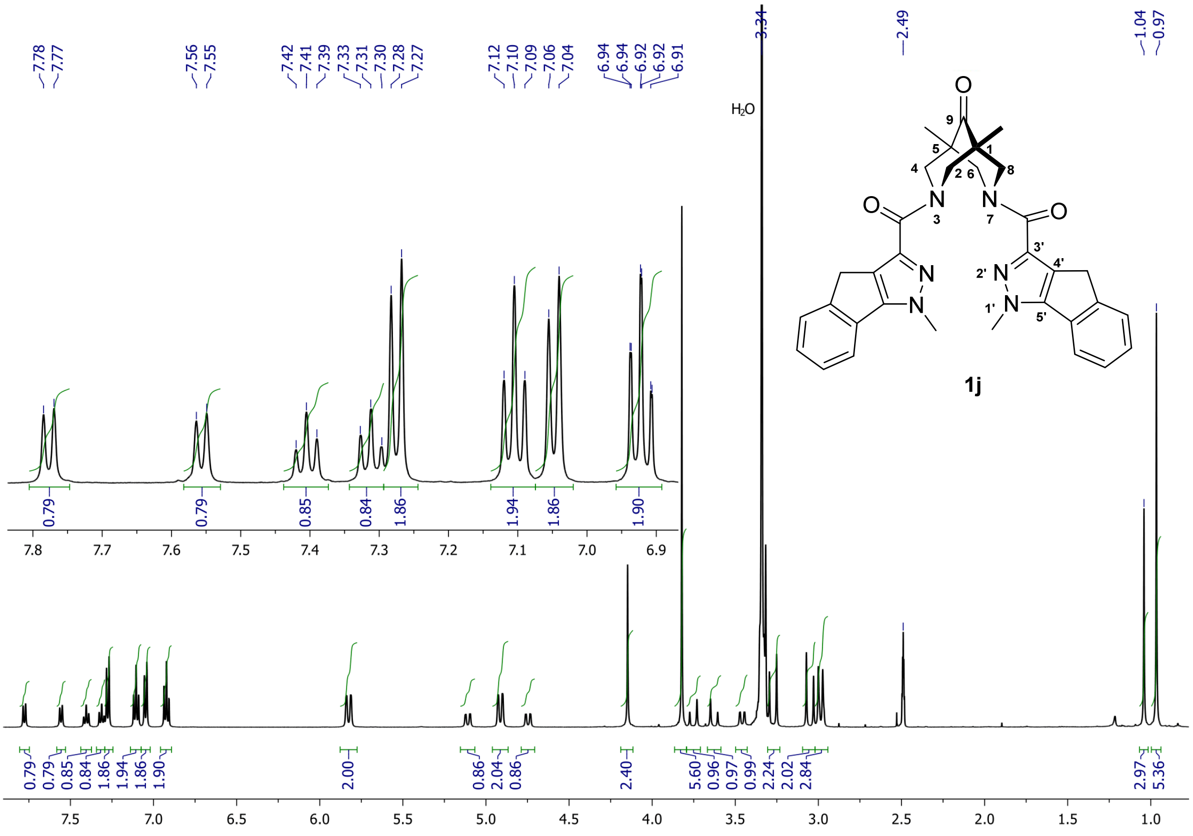

Figure S31. ^{1}H NMR spectrum of **1j** in DMSO-d$_6$.

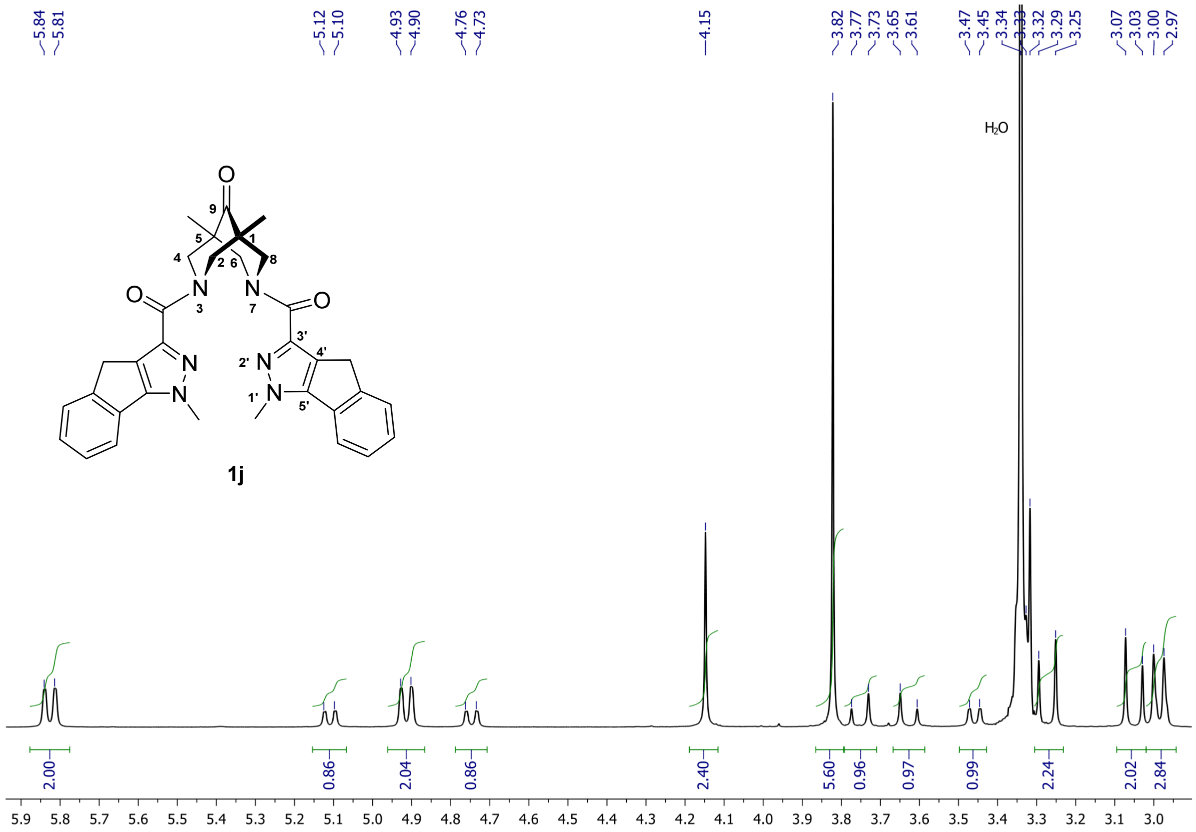

Figure S32. ^{1}H NMR spectrum (aliphatic region) of **1j** in DMSO-d6.

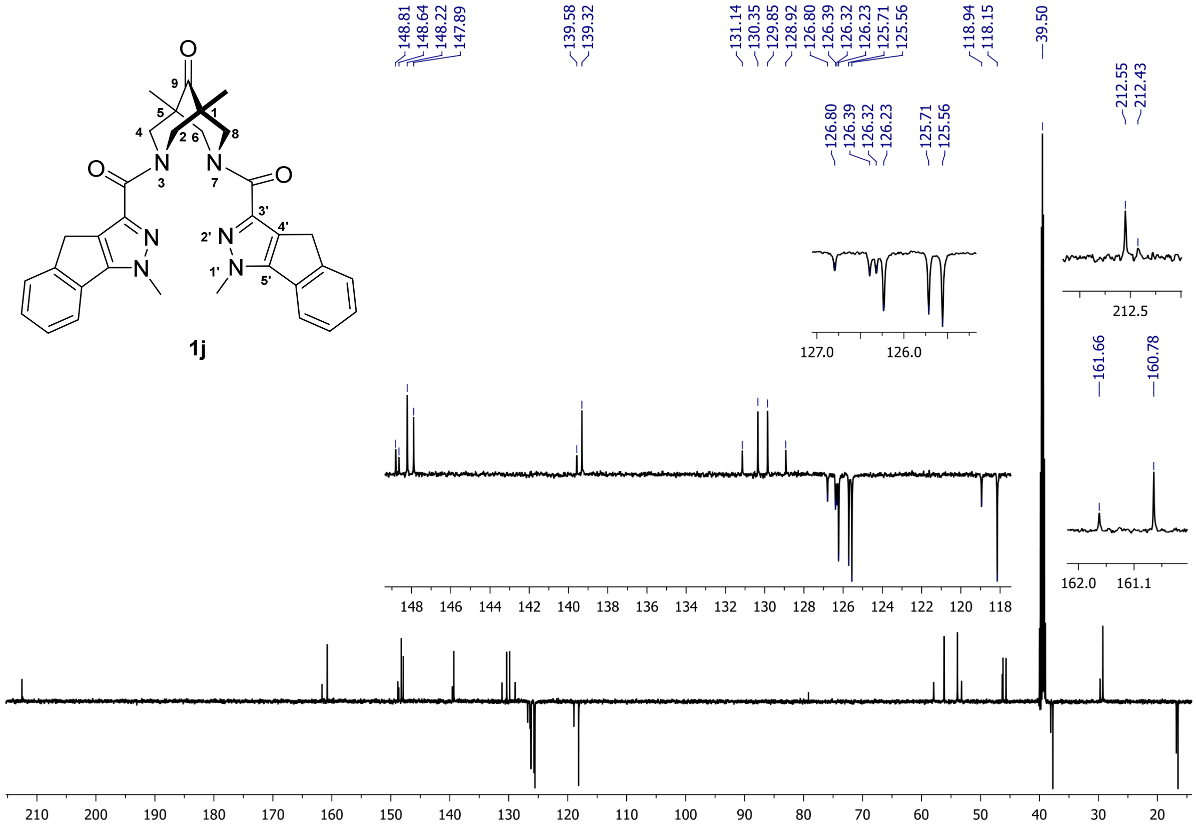

Figure S33. *APT* NMR spectrum of **1j** in DMSO-d6.

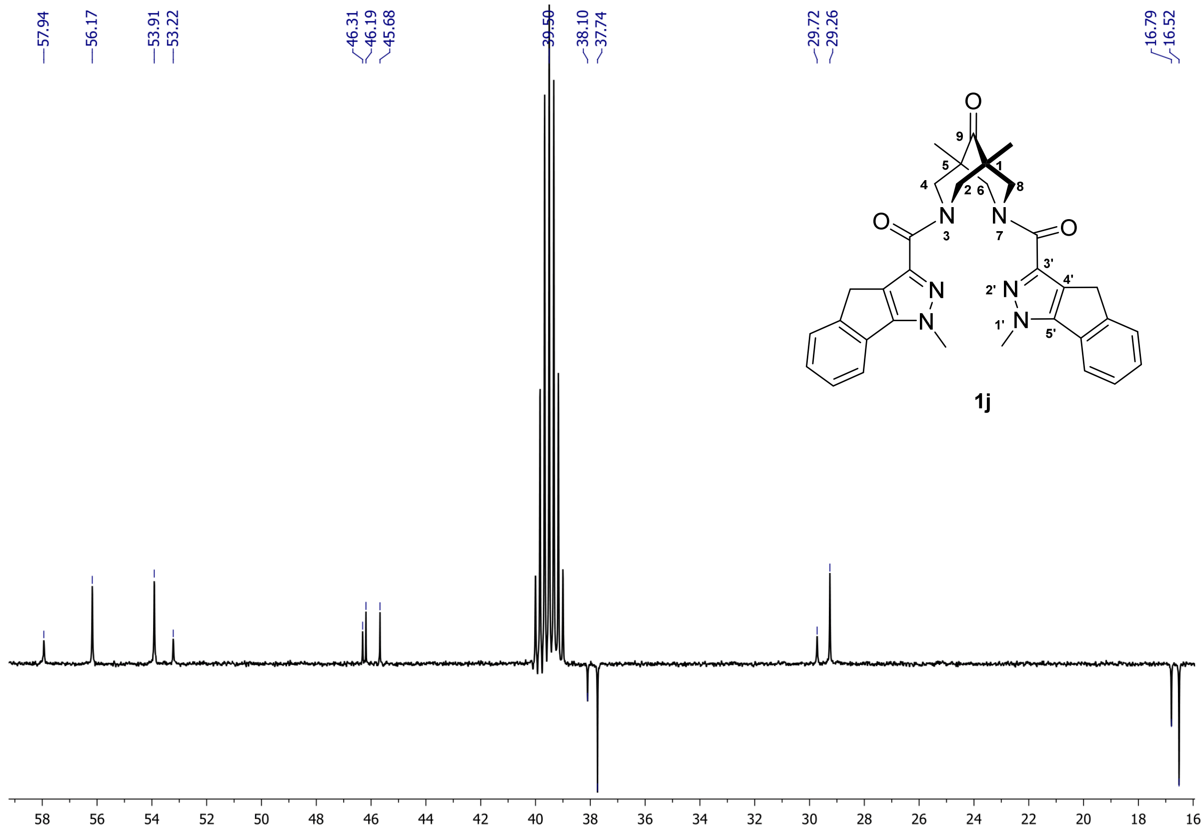

Figure S34. *APT* NMR spectrum (aliphatic region) of **1j** in DMSO-d6.

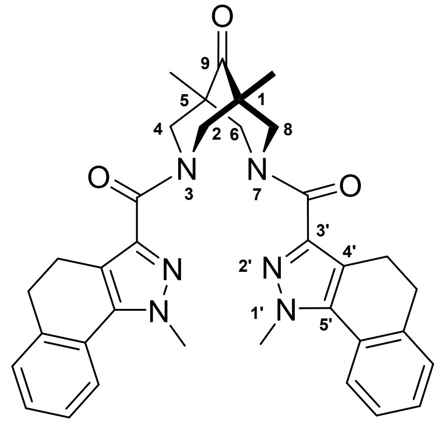

1k

Compound **1k** (*syn-*/*anti-* = 2.40). ^{1}H NMR (500 MHz, $(CD_3)_2SO$, 25 °C, δ/ppm, *J*/Hz): 0.94, 1.04 (both s, CCH_3 (*syn-*)); 0.98 (s, CCH_3 (*anti-*)); 2.25 – 2.82 (set of m, CH_2CH_2 (*syn-*, *anti-*)); 2.98 (d, $^2J_{HH}$ = 13.3, H(ax) (*syn-*)), 2.99 (d, $^2J_{HH}$ = 13.5, H(ax) (*anti-*)); the doublet of second H(ax) (*syn-*)) is overlapped with the signal of H_2O; 3.42 (d, $^2J_{HH}$ = 13.5, H(ax) (*anti-*)); 3.84 (s, NCH_3 (*syn-*)); 4.16 (s, NCH_3 (*anti-*)); 4.84, 5.22 (both dd, $^2J_{HH}$ = 13.5, $^4J_{HH}$ = 2.3, H(eq) (*anti-*)); 4.88 (dd, $^2J_{HH}$ = 13.3, $^4J_{HH}$ = 2.2, H(eq) (*syn-*)); 5.53 (dd, $^2J_{HH}$ = 13.4, $^4J_{HH}$ = 2.2, H(eq) (*syn-*)); 7.11 – 7.38 (set of m, H(Ar) (*syn-*, *anti-*)); 7.73 (d, $^3J_{HH}$ = 7.6, H(Ar) (*anti-*)). ^{13}C NMR (125 MHz, $(CD_3)_2SO$, 25 °C, δ/ppm): 16.53,16.79 (CCH_3 (*syn-*)); 16.68 (CCH_3 (*anti-*)); 19.24, 29.30 (CH_2CH_2 (*syn-*)); 19.60, 29.86 (CH_2CH_2 (*anti-*)); the signals of NCH_3 (*syn-*, *anti-*) are

overlapped with the signal of $(CD_3)_2SO$; 45.76, 45.97 ($C^{1,5}$ (*syn*-)); 46.06 ($C^{1,5}$ (*anti*-)); 53.42, 57.61 (CH_2N (*anti*-)); 53.70, 56.52 (CH_2N (*syn*-)); 121.25, 126.29 (C(Ar) (*anti*-)); 121.45, 125.93 (C(Ar) (*syn*-)); 121.87, 126.68, 127.53, 128.58 (CH(Ar) (*syn*-)); 122.51, 126.96, 127.79, 128.79 (CH(Ar) (*anti*-)); 136.79, 137.42, 140.10 ($C^{3',4',5'}$ (*syn*-)); 137.18, 137.84, 140.37 ($C^{3',4',5'}$ (*anti*-)); 161.50 (C(O)N (*syn*-)); 161.62 (C(O)N (*anti*-)); 212.69 (C^9=O (*syn*-)); 212.73 (C^9=O (*anti*-)).

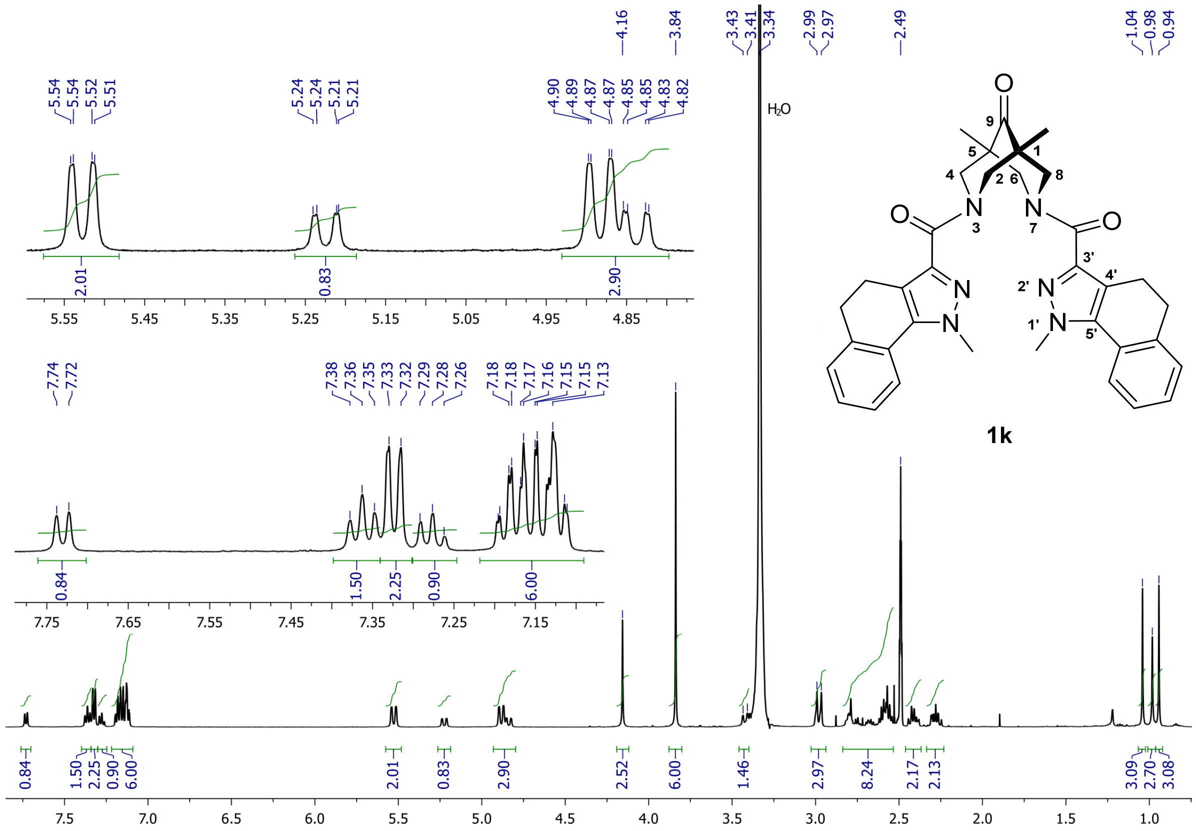

Figure S35. ^{1}H NMR spectrum of **1k** in DMSO-d6.

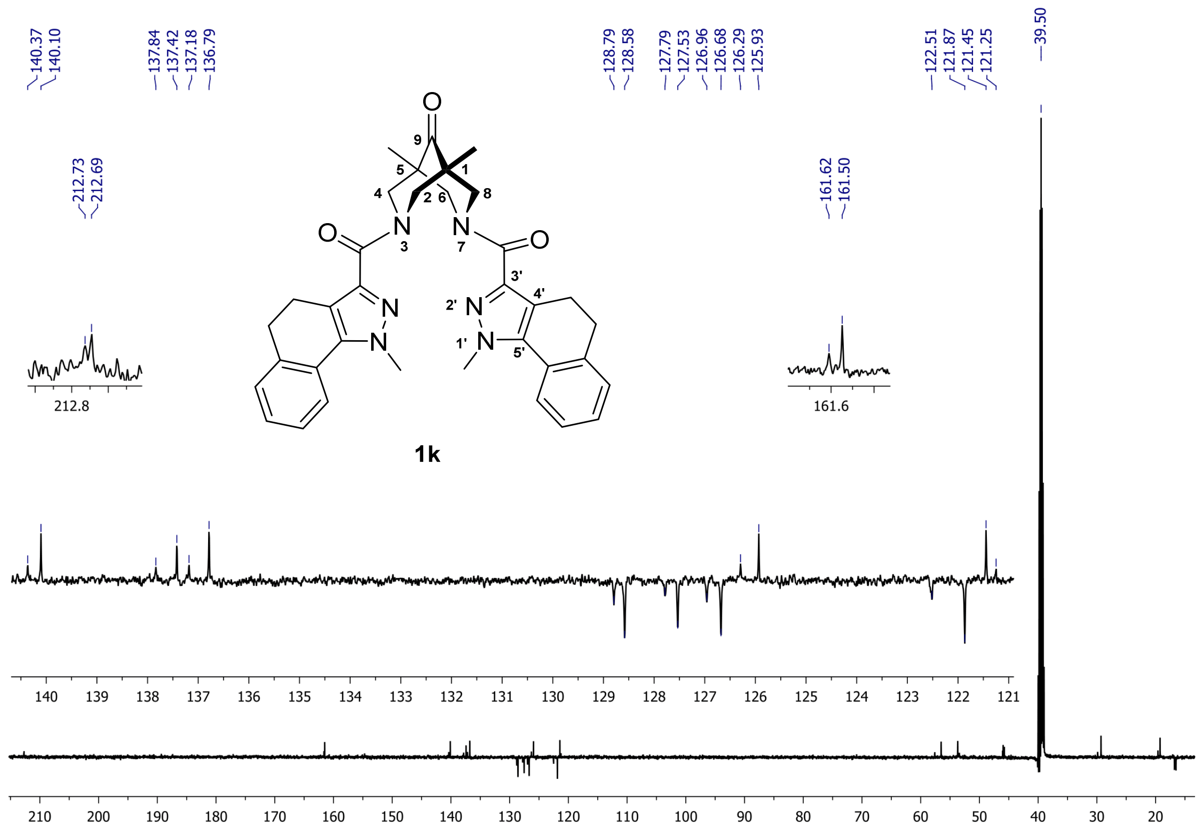

Figure S36. *APT* NMR spectrum of **1k** in DMSO-d6.

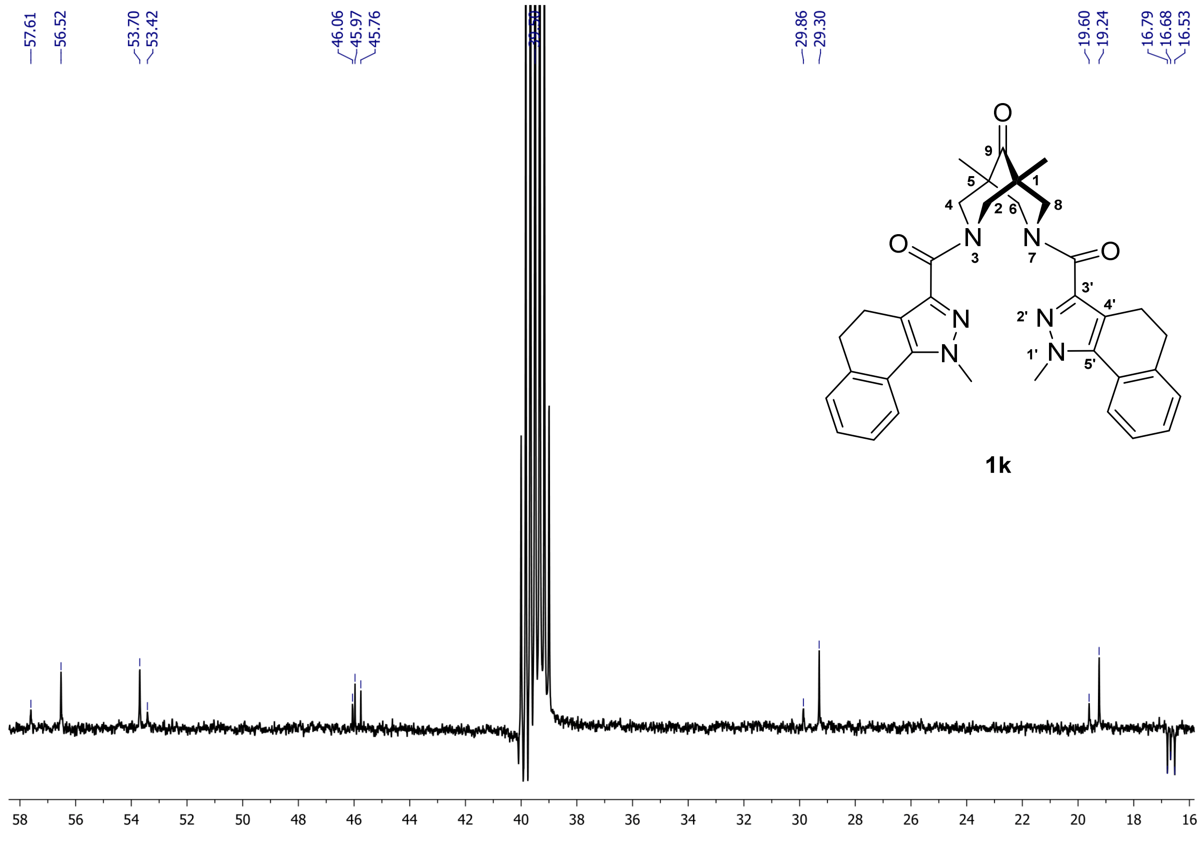

Figure S37. *APT* NMR spectrum (aliphatic region) of **1k** in DMSO-d6.

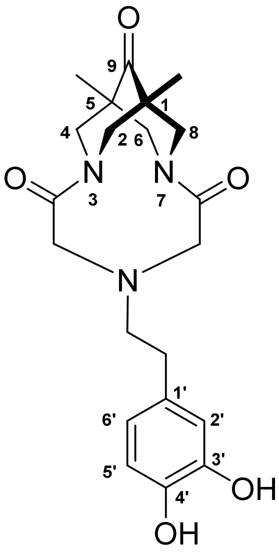

3

Compound **3** (*syn*-). ^{1}H NMR (400 MHz, $(CD_3)_2SO$, δ/ppm, *J*/Hz): 0.70, 0.86 (both s, 3 H, CH_3); 2.65, 2.74 (both m, 2 H, NCH_2CH_2Ar); 2.76 (d, 2 H, $^{2}J_{HH}$ = 13.1, $H^{2,8}$(ax) or $H^{4,6}$(ax)); 2.93 (d, 2 H, $^{2}J_{HH}$ = 14.4, NCH_2(CO)); 2.98 (d, 2 H, $^{2}J_{HH}$ = 13.5, $H^{4,6}$(ax) or $H^{2,8}$(ax)); 3.95 (d, 2 H, $^{2}J_{HH}$ = 14.4, NCH_2(CO)); 4.55 (dd, 2 H, $^{2}J_{HH}$ = 13.1, $^{4}J_{HH}$ = 2.7, $H^{2,8}$(eq) or $H^{4,6}$(eq)); 4.71 (dd, 2 H, $^{2}J_{HH}$ = 13.5, $^{4}J_{HH}$ = 2.7, $H^{4,6}$(eq) or $H^{2,8}$(eq)); 6.52 (dd, 1 H, $^{3}J_{HH}$ = 8.0, $^{4}J_{HH}$ = 2.0, $CH^{6'}$); 6.63 (d, 1 H, $^{3}J_{HH}$ = 8.0, $CH^{5'}$); 6.65 (d, 1 H, $^{4}J_{HH}$ = 2.0, $CH^{2'}$); 8.67, 8.69 (both s, 1 H, ArO*H*). ^{13}C NMR (100 MHz, $(CD_3)_2SO$, δ/ppm): 15.22, 15.98 (CH_3); 31.14 ($NCH_2\mathit{C}H_2Ar$); 44.66, 45.27 ($C^{1,5}$); 53.63, 54.70, 60.16 ($CH_2{}^{2,8}$, $CH_2{}^{4,6}$, NCH_2(CO)); 58.27 ($N\mathit{C}H_2CH_2Ar$); 115.51, 115.99, 119.20 ($CH^{2',5',6'}$); 130.37 ($C^{1'}$); 143.55, 145.21 ($C^{3',4'}$); 168.02 (C(O)N); 211.21 (C^{9}=O).

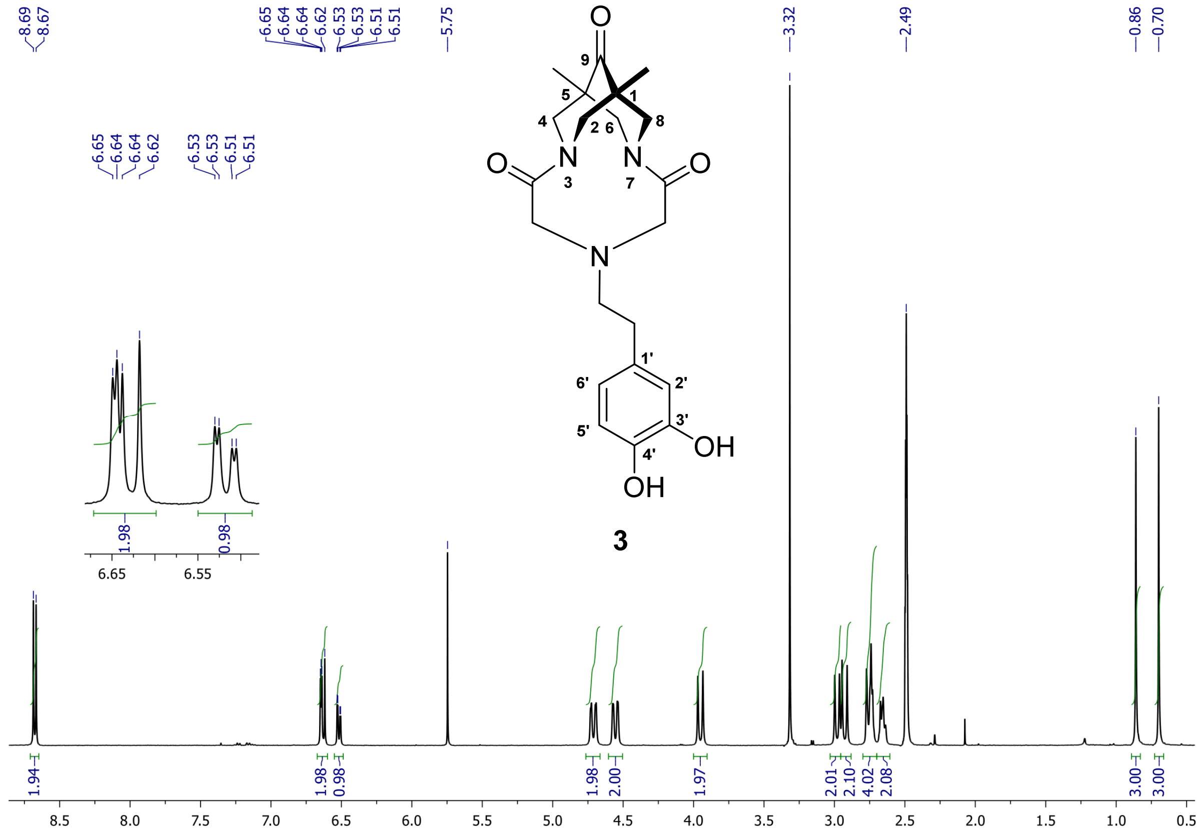

Figure S38. ^{1}H NMR spectrum of **3** in DMSO-d6.

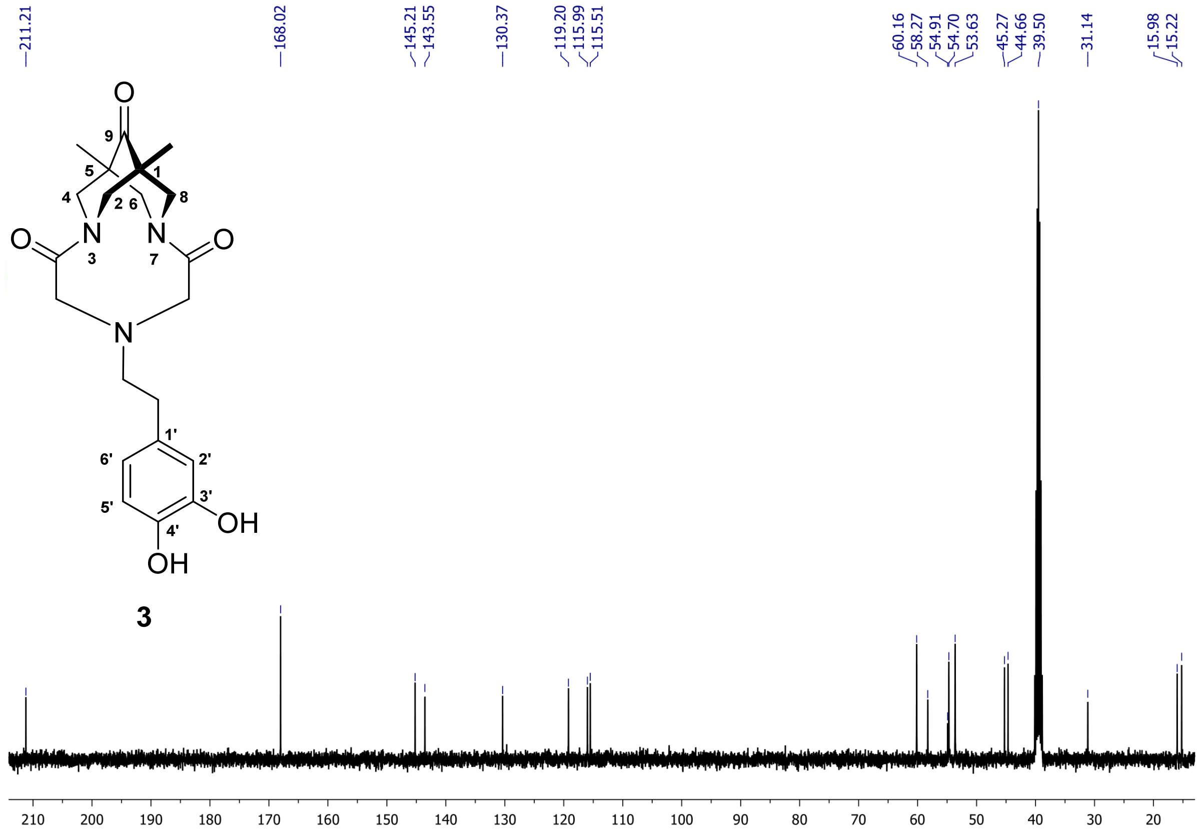

Figure S39. ^{13}C NMR spectrum of **3** in DMSO-d6.

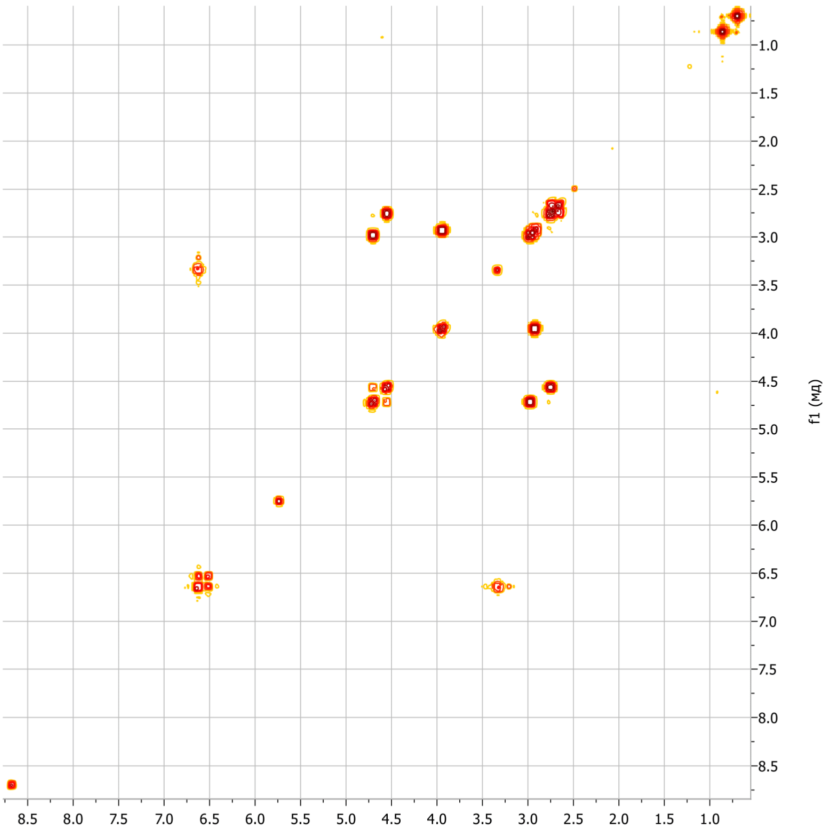

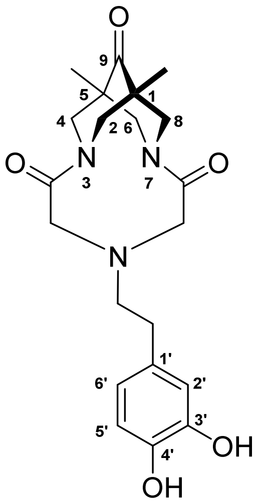

3

Figure S40. *COSY* NMR spectrum of **3** in DMSO-d6.

Quantum chemistry calculations were performed with the Gaussian 09, Revision D.01 program [4] using the density functional theory (PBE0) [5] and the def-2-TZVP basis set. As convergence criteria, the extremely tight threshold limits were applied for the maximum force and displacement. To enhance PBE0 calculation accuracy and increase the reliability of low frequency mode the pruned (99,590) grid (keyword Grid=Ultrafine) has been used. The optimization at PBEO level of theory was followed by the evaluation of the harmonic vibration frequencies.

Topological analysis of the ρ($\boldsymbol{r}$) function, calculations of the v($\boldsymbol{r}_{bcp}$) and integration over interatomic zero-flux surfaces were performed using the AIMAll program [6]. All expected critical points were found and the whole set of critical points in each system satisfies the Poincaré-Hopf rule.

References

1. Churakov, A. V; Medved'ko, A. V; Prikhodchenko, P. V; Krut'ko, D. P.; Vatsadze, S. Z. First example of peroxosolvate of iodine-containing organic molecule. *Mendeleev Communications* **2021**, *31*, 352–355, doi:https://doi.org/10.1016/j.mencom.2021.04.023.
2. Medved'ko, A. V; Krut'ko, D. P.; Gaisen, S. V; Churakov, A. V; Minyaev, M. E.; Moiseeva, A. A.; Lemenovsky, D. A.; Yu, H.; Wang, L.; Vatsadze, S. Z. First examples of bispidine-ferrocene cyclophanes. *Journal of Organometallic Chemistry* **2021**, *949*, 121945, doi:https://doi.org/10.1016/j.jorganchem.2021.121945.
3. Shcherbakov, D.; Baev, D.; Kalinin, M.; Dalinger, A.; Chirkova, V.; Belenkaya, S.; Khvostov, A.; Krut'ko, D.; Medved'ko, A.; Volosnikova, E.; Sharlaeva, E.; Shanshin, D.; Tolstikova, T.; Yarovaya, O.; Maksyutov, R.; Salakhutdinov, N.; Vatsadze, S. Design and Evaluation of Bispidine-Based SARS-CoV-2 Main Protease Inhibitors. *ACS Medicinal Chemistry Letters* **2021**, acsmedchemlett.1c00299, doi:10.1021/acsmedchemlett.1c00299.
4. M. J. Frisch, G. W. Trucks, H. B. Schlegel, G. E. Scuseria, M. A. Robb, J. R. Cheeseman, G. Scalmani, V. Barone, G. A. Petersson, H. Nakatsuji, X. Li, M. Caricato, A. Marenich, J. Bloino, B. G. Janesko, R. Gomperts, B. Mennucci, H. P. Hratchian, J. V. Ort, and D. J. F. Gaussian 09, Revision D.01 2016.
5. Perdew, J. P.; Ernzerhof, M.; Burke, K. Rationale for mixing exact exchange with density functional approximations. *The Journal of Chemical Physics* **1996**, *105*, 9982–9985, doi:10.1063/1.472933.
6. Todd A. Keith AIMAll (Version 19.10.12) 2019.
